# Antimicrobial Peptide K11 Selectively Recognizes Bacterial Biomimetic Membranes and Acts by Twisting Their Bilayers

**DOI:** 10.3390/ph14010001

**Published:** 2020-12-22

**Authors:** Francisco Ramos-Martín, Claudia Herrera-León, Viviane Antonietti, Pascal Sonnet, Catherine Sarazin, Nicola D’Amelio

**Affiliations:** 1 Unité de Génie Enzymatique et Cellulaire UMR 7025 CNRS, Université de Picardie Jules Verne, 80039 Amiens, France; claudia.herrera@u-picardie.fr (C.H.-L.); catherine.sarazin@u-picardie.fr (C.S.); 2 Agents Infectieux, Résistance et Chimiothérapie, AGIR UR 4294, Université de Picardie Jules Verne, UFR de Pharmacie, 80037 Amiens, France; viviane.silva-pires@u-picardie.fr (V.A.); pascal.sonnet@u-picardie.fr (P.S.)

**Keywords:** antimicrobial peptide, biomembranes, ESKAPE, antibiotic resistance, NMR, molecular dynamics, biophysics, sequence alignment

## Abstract

K11 is a synthetic peptide originating from the introduction of a lysine residue in position 11 within the sequence of a rationally designed antibacterial scaffold. Despite its remarkable antibacterial properties towards many ESKAPE bacteria and its optimal therapeutic index (320), a detailed description of its mechanism of action is missing. As most antimicrobial peptides act by destabilizing the membranes of the target organisms, we investigated the interaction of K11 with biomimetic membranes of various phospholipid compositions by liquid and solid-state NMR. Our data show that K11 can selectively destabilize bacterial biomimetic membranes and torque the surface of their bilayers. The same is observed for membranes containing other negatively charged phospholipids which might suggest additional biological activities. Molecular dynamic simulations reveal that K11 can penetrate the membrane in four steps: after binding to phosphate groups by means of the lysine residue at the N-terminus (anchoring), three couples of lysine residues act subsequently to exert a torque in the membrane (twisting) which allows the insertion of aromatic side chains at both termini (insertion) eventually leading to the flip of the amphipathic helix inside the bilayer core (helix flip and internalization).

## 1. Introduction

The persistent use of antibiotics, self-medication and exposure to nosocomial infections has provoked the emergence of multidrug resistant (MDR) bacteria worldwide [1,2,3,4]. The term “ESKAPE” was adopted to refer to some of the most relevant pathogens associated with the highest risk of mortality by the World Health Organization (WHO) [5], namely *Enterococcus faecium*, *Staphylococcus aureus*, *Klebsiella pneumoniae*, *Acinetobacter baumannii*, *Pseudomonas aeruginosa* and *Enterobacter* spp.

In the quest for new molecules able to overcome this major health issue, antimicrobial peptides (AMPs) are promising alternatives to classical antibiotics, due to their low tendency to resistance [6]. AMPs are natural peptides found in all life kingdoms which can be considered components of the innate immunity against bacteria but also fungi, parasites, virus and cancer. Their reduced tendency to resistance is intrinsically due to their mechanism of action causing the selective disruption of bacterial membranes by acting on the lipidic organization of membranes whose lipid composition cannot be changed by a simple point mutation. While exceptions exist [7], their efficacy is proven by the fact that they have been evolutionarily optimized over millions of years, their fast killing rate discourages the rise of drug-resistant mutants [8] and horizontal transfer of resistance genes against AMPs is infrequent [9]. As opposed to standard antibiotics, many AMPs are able to rapidly permeate bacteria and cause irreversible damage to their cell membranes, leading to the death of microorganisms [10,11]. In some cases, their action is also intracellular [12,13].

Several AMPs have been rationally optimized and in this work we focus on K11, a synthetic AMP which was reported to exert antimicrobial action against many of the mentioned ESKAPE bacteria such as *Acinetobacter baumannii*, methicillin-resistant *Staphylococcus aureus*, *Pseudomonas aeruginosa*, *Staphylococcus epidermidis*, and *Klebsiella pneumoniae* [14,15]. K11 has also been successfully used in-vivo as a topic hydrogel solution against *A. baumannii*-infected wounds [15]. Its mechanism of action deserves special attention considering that many of its bacterial targets [14,15] cause complex infections because of their ability to form biofilms [16,17,18] or change their membrane composition. For example, *A. baumannii* is not only able to form biofilms on biotic and abiotic surfaces but it can also develop resistance to colistin by incorporating phospholipids such as phosphatidylethanolamine (PE), cardiolipin (CL) and monolysocardiolipin to remodel its lipid composition [18,19].

From the point of view of the sequence, K11 (KWKSFIKKLTKKFLHSAKKF-NH2) is an example of synthetic peptide inspired by natural AMPs (cecropin A1, melittin and magainin) [14,15]. More specifically, K11 is one member of a group of peptides synthesized from the CP-P designed antibacterial scaffold (KWKSFIKKLTSKFLHLAKKF). This template was created [14] from the N-terminus of CP26 peptide (inspired by cecropin A1 and melittin) and C-terminus from P18 peptide (inspired by cecropin A1 and magainin) [20]. While CP26 has been reported to target bacterial lipopolysaccharides (LPS) [21], P18 also displays anticancer activity [20]. Most importantly, both CP26 and P18 display antimicrobial activity and negligible toxicity. The introduction of a lysine in position 11 in the CP-P template (hence the name) led to the K11, a peptide with improved values of the therapeutic index (320) [14]. It is believed that the introduction of lysine 11, besides changing the net positive charge, would also alter its amphipathic structure. However, more structural studies are needed to elucidate its mode of action [14].

The interesting properties of K11 prompted us to investigate its interaction with biomimetic membranes by liquid and solid-state NMR spectroscopy (ssNMR) and Molecular Dynamic simulations (MD). Nowadays many different lipidic systems have been optimized for such kinds of studies, going from dodecylphosphocholine (DPC) micelles to bicelles and liposomes with variable phospholipid and sterol compositions reproducing those of the target organisms. The membrane of K11 bacterial targets contains PE, phosphatidylglycerol (PG) and CL in various amounts, as most bacteria. In particular, *Pseudomonas aeruginosa* [22], *Escherichia coli* [23], *Salmonella paratyphi* [24], *Acinetobacter baumannii*, and *Klebsiella pneumoniae* are rich in PE, as expected for the outer membrane of many gram-negative bacteria [25]. Some of its gram-positive targets such as *Bacillus subtilis* and *Bacillus pumilus*, contain PE and PG (although the distribution of phospholipids is unclear) [26,27,28], while PG or CL clearly prevail in others, such as *Staphylococcus epidermidis* [29,30], *Staphylococcus aureus* [25] and *Micrococcus luteus* [31]. Independently of the relative composition of PG and PE, a special network of H-bond or water-bridged interactions can be established between the two phospholipids [25,32,33], whose ratio can be modulated by bacteria in response to external agents or conditions [22,28,34]. For example, *S. aureus* and *S. epidermidis* can increase their amount of CL under high salt conditions [29,30].

In this work, we show that K11 is able to penetrate biomimetic membranes reproducing the phospholipid composition found in bacteria. Most intriguing, we show that the peptide might act by twisting the membrane using couples of lysine residues. According with this mechanism, the introduction of lysine 11 (whose introduction in the related CP-P peptide significantly improved the therapeutic index) would act in couple with lysine 12 and synergically with all other lysines to torque the membrane, thus facilitating the insertion of aromatic residues at both termini (phenylalanine or tryptophan) and eventually the full peptide in the innermost part of bacterial bilayers. Additionally, for the first time we have observed an interaction with phosphatidylserine (PS), a phospholipid often involved in a wide range of biological processes including viral infection and carcinogenesis [35,36,37]. 

## 2. Results and Discussion

The work was organized as follows. First, property-alignment [38] was used to highlight important motifs along the sequence and to explore further possible activities of K11. Second, we studied the structure of the peptide in solution and in the presence of simple biomimetic models (micelles and isotropic bicelles). Third, ssNMR was used to characterize the effect of K11 on the lipid assembly in vesicles containing various compositions of PE, PG and CL, due to their importance in bacterial membranes. In the attempt to understand the selectivity and low toxicity of K11, membranes containing phosphatidylcholine (PC) were used to mimic the outer leaflet of eukaryotic cells. PS was also considered to explain further predicted activities. Finally, the studied systems including an even larger variety of phospholipids were studied by molecular dynamic simulations for a deeper understanding of the experimental results and of the mechanism of action.

### 2.1. Property-Sequence Alignment of K11 Highlight Antibacterial Motifs and Predicts Further Activities

In order to highlight structure-function relations, we performed property-alignment in the ADAPTABLE web server [38]. It is important to point out that property alignment clusters sequences with specific activities (antibacterial in this case). The K11 peptide is one of a series of synthetic peptides obtained by designed mutations of the CP-P template [14]. All of them are present in the ADAPTABLE database but they are not meaningful when evaluating the importance of conserved residues among evolutionary-distant sequences. Excluding these entries (peptides 2–22), the sequence-related family (SR family) (Figure S1A) shows that the KWK motif at the N terminus and a large portion of the C-terminus seem to be recurrent in peptides with antibacterial activity. Interestingly, eight out of nine meaningful sequences exhibit activity towards a large variety of cancers [39] (Figure S1B). Such predicted activity could be explained by the fact that one of K11 precursors, P18 has also anticancer properties [20]. One related peptide exhibits antifungal activity against *Candida albicans* and *Trichosporon beigelii* [40]. It should be noted that PS plays a relevant role in both cancer development [35,41,42,43] and *Candida albicans* virulence [44,45,46,47,48] for which PG [49] and PI [47] are also important.

### 2.2. K11 Peptide Is Unstructured in Aqueous Solution 

**Figure 1:**
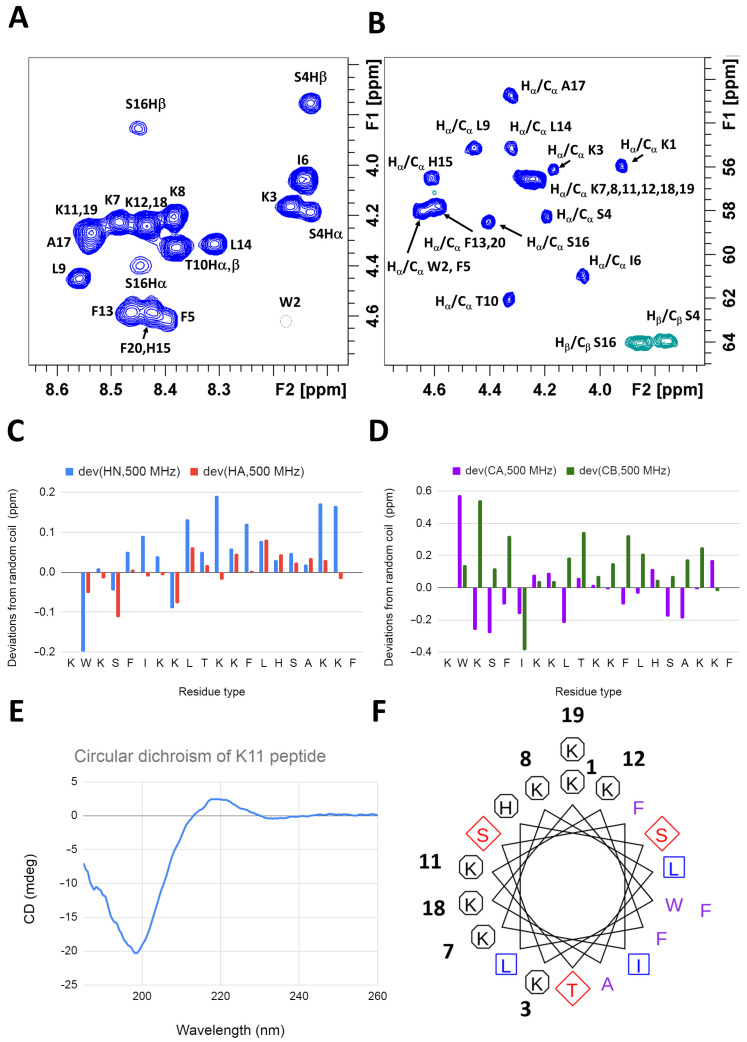
(**A**,**B**) The ^1^H and ^13^C NMR assignment of K11 on the amide/Hα (**A**) and side chain (**B**) regions of ^1^H, ^1^H-TOCSY and ^1^H,^13^C-HSQC spectrum, respectively. (**C**,**D**) Chemical shift deviations from random coil values of amide HN and Hα protons (**C**) and Cα and Cβ carbons (**D**); (**E**) CD spectrum of K11 in solution; (**F**) helical wheel showing the disposition of side chains in alpha helical conformation.

The ^1^H and ^13^C NMR assignment of K11 is reported in Table S1 and Figure 1A,B. The deviations from random coil values [50,51,52] indicate that the peptide is mainly unstructured in solution (Figure 1C,D), as also confirmed by circular dichroism (CD) (Figure 1E). The formation of a stable helix (theoretical helical wheel in Figure 1F), which would approach many positive charges arising from eight lysine residues, is probably disfavored in the absence of charge-compensating molecular partners.

#### 2.2.1. K11 Peptide Assumes Alpha Helical Conformation in a Lipidic Environment

**Figure 2:**
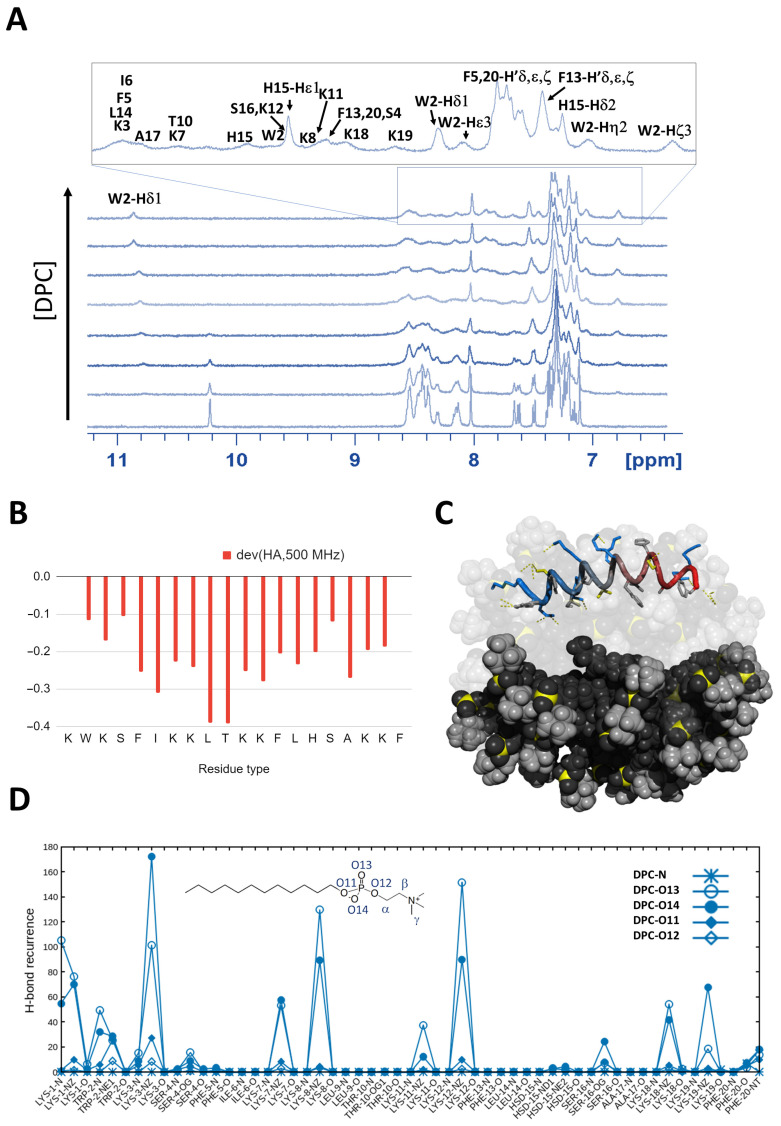
(**A**) ^1^H NMR spectra of K11 1 mM in the presence of DPC at concentrations 1, 2, 4, 10, 20, 30, 60 mM. The NMR assignment in the presence of micelles is also shown. (**B**) Chemical shift deviations from random coil values of Hα protons whose negative deviations indicate an alpha helical conformation; (**C**) MD snapshot of K11 interacting with DPC micelles; (**D**) Polar contacts recurrence (H-bonds and salt bridges) along MD simulation.

The titration of K11 with a concentrated solution of DPC induces drastic changes in the ^1^H NMR spectrum as shown in Figure 2A. New peaks appear in the spectrum while the signals originating from the unbound peptide gradually disappear as the concentration of DPC increases. The slow exchange regime is clearly exemplified by the isolated signal of W2 Hδ1, disappearing at its original shift of 10.2 ppm and reappearing at 10.8 ppm (see Figure 2A). A closer analysis of ^1^H,^13^C-HSQC spectrum (Figure S2A–D), reveals that all aromatic residues (Figure S2A,B) are deeply affected by the presence of the micelles with the exception of H15, whose signals only slightly shift and partially lose intensity (see Figure S2A). A significant shift is also observed for aliphatic signals of F5, I6, L9, T10, F13, L14, A17, F20 (Figure S2C,D) which would be located on the same molecular face in case an alpha helix is formed. A clear proximity of the aromatic (Figure S2E) and aliphatic (Figure S2F) side chains to the DPC acyl chains is demonstrated by the NOE cross peaks in the NOESY spectrum. NOEs with the acyl chain of DPC (whose assignment was based on the literature [53]) but not with its choline headgroup testify a rather deep insertion of the peptide into the micelle and the absence of a specific interaction with the headgroup. While most ^13^C backbone signals are lost in the ^1^H,^13^C-HSQC spectrum, we were able to assign all Hα protons. Their values were used to predict the secondary structure [50,51,52] of the peptide bound to DPC micelles. The negative deviations from theoretical random coil values unequivocally indicate that the peptide assumes an alpha helical conformation (see Figure 2B and Figure 1C for comparison). Accordingly, all the weak HN_i_/HN_i−1/i+1_ NOEs observed in the free peptide gain in intensity (data not shown). All-atom MD simulations are in perfect agreement with NMR data (Figure 2C), showing how the peptide conserves its alpha helical conformation along the full trajectory of 500 ns.

The radial distribution function [54] of each oxygen and nitrogen atom of the membrane from all O/N atoms of the peptide can be used to highlight key interatomic interactions. By focusing on its maximum value in the distance range of H-bond and salt bridges, we obtain a quantification of the frequency at which these interactions occur all along the last 250 ns of the simulations. Results are shown in Figure 2D, revealing that lysine side chains are able to recognize the phosphate groups of the phospholipids. Interestingly, only K1, 3, 8 and 12 interact frequently while K7, 11, 18 and 19 seem slightly less involved (Figure S3). While K7 and 11 might not be at the optimal distance, the absence of interaction of K19 might be due to the high curvature of the micelle, not allowing all lysines of the helix to interact at the same time.

#### 2.2.2. In the Presence of Biomimetic Bicelles K11 Peptide Possibly Assumes a Conformation Similar to That Found with Micelles

In order to better understand the influence of the curvature, we studied the interaction of K11 peptide with isotropic bicelles, better representing a more extended surface in solution [55]. Isotropic bicelles can be formed by a mixture of DMPC (1,2-dimyristoyl-sn-glycero-3-phosphocholine) and DHPC (1,2-dihexanoyl-sn-glycero-3-phosphocholine) [56]. The short acyl chain of DHPC is able to stabilize the bilayer formed by DMPC, whose myristoyl hydrophobic chains would be otherwise exposed to the solvent [57]. Fast tumbling isotropic bicelles can be obtained at a DMPC/DHPC ratio 1:2 (*q* = 0.5). As in the case of micelles, the ^1^H NMR spectrum of K11 peptide drastically changes in the presence of bicelles (70 mM) but NMR signals become too broad for a new assignment. However, the NMR spectrum resembles the one observed in the presence of micelles (see Figure S4) suggesting that the same helical conformation is formed. 

In DMPC/DHPC bicelles part of DMPC lipids can be substituted by phospholipids with different headgroups to mimic different biological membranes [57]. In this way, bicelles containing PE, PG, and PS were formed and tested in their interaction with K11. Figure S4 shows that, although the spectra are qualitatively similar, the linewidth is significantly larger in the case of DMPG (1,2-dimyristoyl-sn-glycero-3-phospho-(1′-rac-glycerol)) and DMPS (1,2-dimyristoyl-sn-glycero-3-phospho-l-serine), probably indicating that the peptide is less mobile inside these bilayers because of a stronger interaction. Interestingly, both DMPG and DMPS introduce negative charges in the bicelles which might stabilize the structure of the positively charged K11 peptide.

#### 2.2.3. K11 Selectivity Perturbs the Core of Liposomes with Bacterial Phospholipid Compositions

In order to ascertain the effect of K11 on the lipid assembly of the membrane, we studied the interaction of K11 with multilamellar vesicles (MLVs) by ssNMR. 

**Figure 3:**
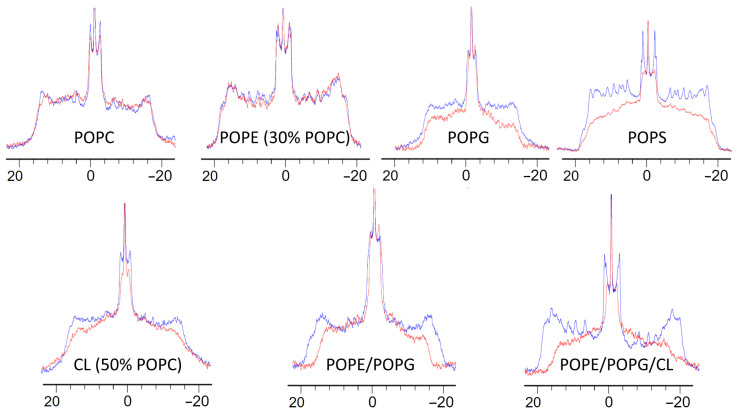
Static ^2^H NMR spectra of various multilamellar vesicles (MLVs) in the absence (blue) and in the presence (red) of K11 peptide.

MLVs are more suitable to mimic lipid bilayers of biological membranes because of their hydration state and the lower curvature than bicelles. They also allow to vary the phospholipid composition more freely [55] while bicelles always require a large part of DMPC and DHPC. Moreover, MLVs can be prepared by using commercially available phospholipids bearing deuterated palmitic chains allowing to sense the organization of the hydrophobic core of the lipid bilayer by ^2^H NMR. Thus, the order parameter for each C-^2^H bond of the chain can be measured by means of ^2^H quadrupolar splitting [58,59,60]. Figure 3 shows ^2^H spectra of MLVs with various phospholipid compositions and the effect of the presence of K11. Each spectrum results from a superposition of the quadrupolar doublet arising from different C-^2^H bonds. Since the mobility of unsaturated chains in bilayers increases as we move away from the headgroup, the quadrupolar splitting also decreases, with the consequence that methyl groups appear at the center of the envelope while the carbon in position 2 appears at the extremities of the spectrum, with all the remaining C-^2^H in-between. Even though we could not measure the order parameters for each C-H moiety because of the low resolution, the overall behavior is very clear when observing the width of the superimposed signals. The presence of K11 does not perturb the ^2^H spectra of POPC (1-palmitoyl-2-oleoyl-glycero-3-phosphocholine) membranes and POPE (30% POPC) (1-palmitoyl-2-oleoyl-sn-glycero-3-phosphoethanolamine), suggesting that the peptide is not able to penetrate deeply into these bilayers. This observation stresses the importance of the curvature in biomimetic models. Despite the presence of the same headgroup, K11 deeply penetrates DPC micelles but not POPC MLVs, where lipids are more closely compacted because of the locally almost planar surface as opposed to the high curvature of micelles [55].

Quite interestingly, K11 deeply affects the ^2^H spectrum of POPG (1-palmitoyl-2-oleoyl-sn-glycero-3-phospho-(1′-rac-glycerol)) and POPS (1-palmitoyl-2-oleoyl-sn-glycero-3-phospho-l-serine) MLVs. These headgroups are commonly found in bacteria and cancer cells, respectively, but also fungi, as hypothesized in the activity prediction based on property-alignment by ADAPTABLE. Our data reproduce qualitatively what was found with bicelles, where the increased linewidth observed with PG and PS headgroups suggested a stronger binding (see Section 2.2.2). For both POPG and POPS MLVs, the apparent reduction of the quadrupolar splitting and the loss of resolution reflects a drastic increase in the phospholipid acyl chain mobility, most probably due to the internalization of the peptide in the bilayers. Encouraged by these results, we prepared MLVs using a mixture of PE and PG headgroups, typically found in bacteria [33]. As shown in Figure 3, K11 is able to perturb the fluidity of such bilayers even more and the effect becomes really important in the presence of CL, also found in bacteria [61]. Figure 3 shows how K11 affects also membranes mainly constituted by CL (CL 50% / POPC 50%). It should be noted that in the cases of CL and POPE, the addition of POPC was necessary for the formation of a MLV, due to their intrinsic shape and different *T*_m_ [32,62,63,64,65,66,67,68].

### 2.3. MD Simulations Provide a Molecular Picture of the Interaction

**Figure 4:**
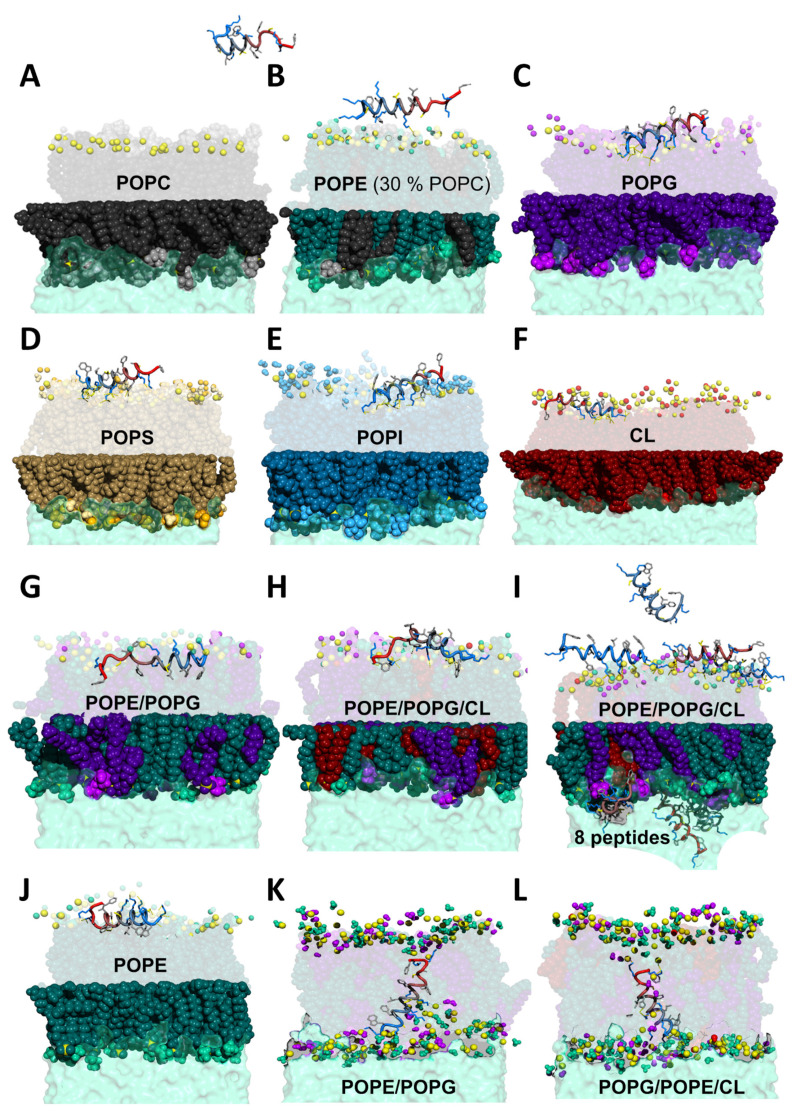
MD snapshots representative of K11 peptide interacting with several membranes of variable phospholipid compositions. Color code: (**A**) POPC black (body) and light gray (choline group); (**B**) POPE dark green (body), turquoise (headgroup) and light green (amine of the headgroup); (**C**) POPG dark violet (body), violet (headgroup) and light violet (hydroxyls of the headgroup); (**D**) POPS brown (body), gold (headgroup), light yellow (amine of the headgroup) and orange (carboxyl of the headgroup); (**E**) POPI blue (body), light blue (headgroup) and cyan (hydroxyls of the headgroup); (**F**) CL dark red (body) and light red (headgroup). Panels G-H show lipid mixtures typically found in bacteria: (**G**) POPE/POPG and (**H**) POPE/POPG/CL. Panel (**I**) represents an example of calculation with eight peptides while panel (**J**) refer to a calculation of one peptide interacting with a pure POPE membrane, differing from that in panel B for the absence of POPC. Snapshots in panels (**K**), (**L**) refer to examples of simulations where K11 is purposely placed inside the membrane at the start of the calculation. In all panels the phosphorus atom of phospholipids is shown as a yellow sphere; for clarity, only functional moieties of headgroups are represented as spheres either in the upper leaflet, or in both leaflets (panels K, L). K11 peptide is shown as a “tube” colored from blue (N-terminus) to red (C-terminus) except in panel I where each of the eight peptide has a different color. Side chains are shown as sticks with the following color code: positively charged (blue), negatively charged (red), non-polar (light gray), polar (yellow).

In order to get insight into the details of the interaction between K11 peptide and biomembranes, we performed MD simulations using a variety of phospholipid combinations involved in bacteria, cancer and fungi. Figure 4 shows the most significant snapshot for each run, and in particular the frames where the peptide comes close to the membranes.

In the case of POPC, K11 stays away from the bilayer during most of the simulation and even when it approaches the membrane, there is no evidence of a specific interaction. For POPE (containing 30% of POPC to reproduce the experimental conditions), the situation is only slightly different. In this case, the peptide can lay on the surface sporadically and very few interactions are established.

The situation is radically different for POPG and in general for all negatively charged phospholipids: POPS, POPI (1-palmitoyl-2-oleoyl-sn-glycero-3-phosphoinositol), and CL. A clearer picture comes from the analysis of polar interactions shown in Figures S5 and S6. As in Section 2.2.1, we calculated the recurrence of H-bond and salt bridges (Figures S5 and S6). In the case of POPG we observe an important interaction of the N-terminal lysine with the oxygen atoms of POPG phosphate groups by means of both the backbone and side chain amines. Such an interaction is consistently observed when the peptide significantly interacts, suggesting that K11 peptide approaches the membranes with the first lysine residue. Furthermore, also the side chains of lysine residues in position 3, 7 and 8 establish similar contacts. The selectivity for negatively charged phospholipids like POPG is probably due to the electrostatic attraction leading the positively charged K11 peptide (+8 at physiological pH) towards the negative charges introduced by POPG headgroup but also to the availability of multiple oxygen atoms provided by its glycerol moiety (inositol and carboxylate in the case of POPI and POPS, respectively), which are available for hydrogen bonding or the formation of extra salt bridges in the case of POPS. Not surprisingly, a strong interaction is also observed with CL due to the structural similarity to POPG, the exposition of phosphate groups at the membrane surface and the doubly negative charge. When PG or CL are in mixtures, they are involved in the large majority of the peptide-membrane interactions (see Figure S6).

The Coulomb attraction is clearly a key factor to understand such selectivity but also other factors play a role: the inter-lipid spacing (modulated by the curvature [69,70]), the steric hindrance of the headgroups and the amount of inter-lipid interactions [34,35]. The first factor explains why K11 can penetrate DPC micelles (whose PC headgroup is not found in bacteria) but doesn’t seem to affect POPC liposomes significantly. In this case, the high curvature makes the inter-lipid spacing wider, facilitating the access to the micelle core. The second factor explains the preference for CL over PE and PG when the three are present (see Figure S6); in the case of CL phosphate moieties are directly accessible as its headgroup doesn’t present steric hindrance. The third factor is also important, because the insertion of the peptide implies breaking a number of favorable interactions (like the ones present in PE/PG membranes [25,32,33]). Besides these effects, the presence of negative charges on the membranes does incentivize the binding not only in the case of PS but also with PG and PI (Figures S5 and S6). In similar conditions of curvature, we can expect that the accessibility to phosphate moieties is modulated by the steric hindrance of headgroups but also the network of inter-lipid interactions. Last but not least, the mobility of the peptide in the complex and the degree of lipid-order destabilization might contribute to the overall process as entropic contribution [71].

#### 2.3.1. K11 Exerts a Twisting Effect of Its Target Membranes

**Figure 5:**
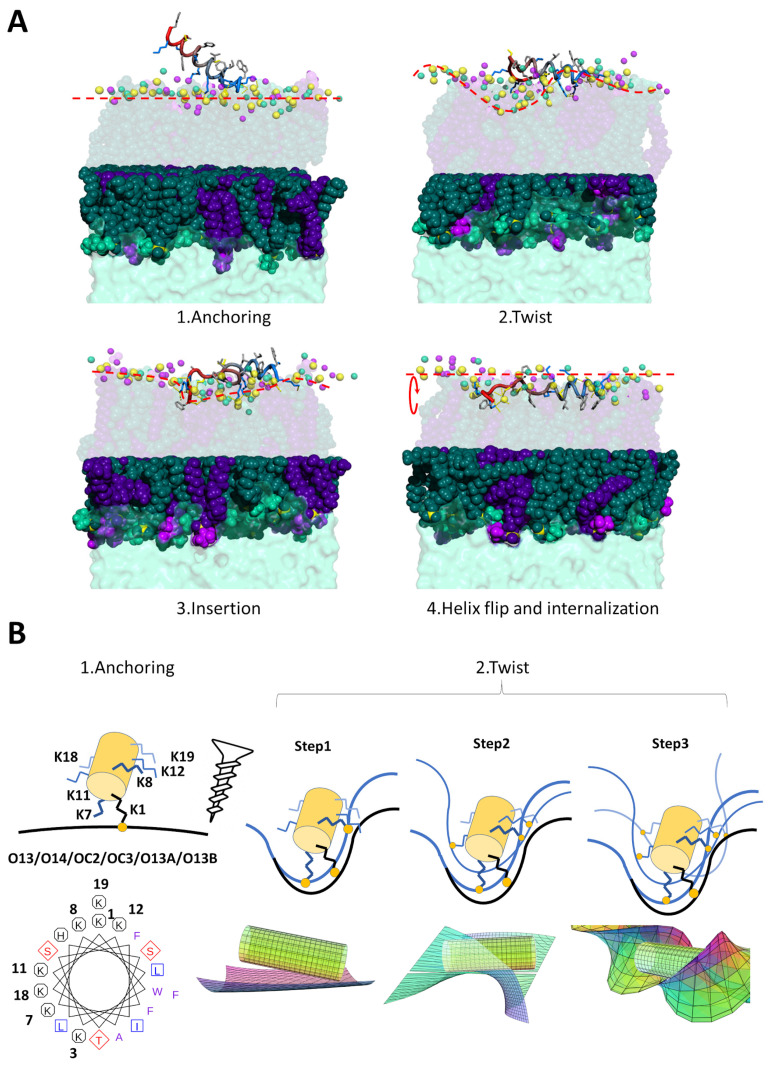
(**A**) Proposed mechanism of action of K11 peptide in four steps. The peptide first anchors to the membrane by the lysine residue in position 1 (anchoring) which can bind membrane oxygen atoms in a bidentate fashion. The peptide then twists the membrane (as described in panel B) thus allowing the insertion of terminal aromatic side chains. Finally, the peptide flips inside the bilayer. For color codes refer to the caption of Figure 4. (**B**) Mechanism by which K11 might exert a torque on the target membrane. Yellow circles represent oxygen atoms on the surface of the membrane and available for H-bonding or salt bridges with lysine side chains. The helix formed by K11 is represented as a cylinder. Lysine residues and membrane planes are represented in blue color whose intensity degrades with the distance from the observer. The torque is achieved in three subsequent steps, each rotating the membrane in the plane described by each couple of lysine residue. A geometrical representation of the effect on the membrane for each step is exemplified under each step. Image generated with the help of CalcPlot3D software [72].

Quite interestingly, the membrane planarity is heavily perturbed and almost twisted when K11 peptide interacts. A closer analysis of the helix wheel reveals that the peptide could act as a screw twisting the membrane by means of couples of lysine residues (Figure 5). In all MD simulations in the presence of membranes containing PG, PS, PI or CL we observe the same behavior (see Figure 5A): the peptide approaches the membrane with the N-terminal lysine (step 1, anchoring), grabs onto the available oxygen atoms (most frequently phosphate oxygen O13 and O14 but also headgroup oxygens) and deforms the membrane (ste p2, twisting). The deformation allows the insertion of terminal aromatic groups (F20 but in some cases also W2) inside the bilayer (step 3, insertion). F20 and W2 are the only amino acids whose hydrophobic side chains are readily available and this is due to the fact that the amphipathic helix formed by K11 approaches the membrane with its hydrophilic face. In particular, in the simulation containing POPE/POPG, we observe a further important step. The insertion of the aromatic ring of F20 eventually determines the flip of the full hydrophobic face of the helix into the bilayer thus reaching the hydrophobic core (step 4, helix flip and internalization). The peptide remains inserted even prolonging the simulation up to 2 μs.

A closer look to the relative disposition of lysine residues in an alpha helix conformation can explain the twisting effect on the membrane (Figure 5B). We believe that K11 peptide actually works as a screw. By landing on the surface with the first lysine, the peptide anchors to the available oxygen atoms. These may arise from the phospholipid phosphate groups or oxygens in the headgroups. Such an anchoring is quite effective because lysine 1 bears two amine moieties that can bind in a bidentate fashion. Figures S5 and S6 shows how such an interaction is present in almost all simulations involving charged phospholipids with high occurrence. We can imagine dissecting the helix of the peptide with three planes almost orthogonal to its long axis, each containing two lysine residues (K7 and 8, K11 and 12, K18 and 19). The K1 anchoring step is followed by the establishment of interactions involving lysines 7 and 8 with available nearby membrane oxygen atoms. These bindings have the synergic effect of rotating the membrane in their plane (see Figure 5B). Such rotation is subsequently reproduced in the plane of lysines 11 and 12 and in that of lysines 18 and 19. As the couples of lysines 7–8, 11–12 and 18–19 are located with different phases in the helix wheel, these subsequent rotations have the effect of a twist. In particular, while lysines 7–8 and 11–12 would determine a clockwise rotation, lysines 18–19 would act in the opposite sense because of their intermediate phase in the wheel.

Significant perturbation of the membrane can be more easily visualized by monitoring the area per lipid along the MD trajectories. Although the perturbation can be detected in simulations with one peptide, the effect is amplified with the introduction of several peptides (see Figure S7). With the exception of POPC and POPE, we observe a decrease in area per lipid of the upper leaflet and an increase in that of the lower leaflet, indicating that the peptide exerts a pressure causing the membrane to invaginate (negative curvature).

#### 2.3.2. K11 First Rigidifies the Membrane and Subsequently Makes It More Fluid 

**Figure 6:**
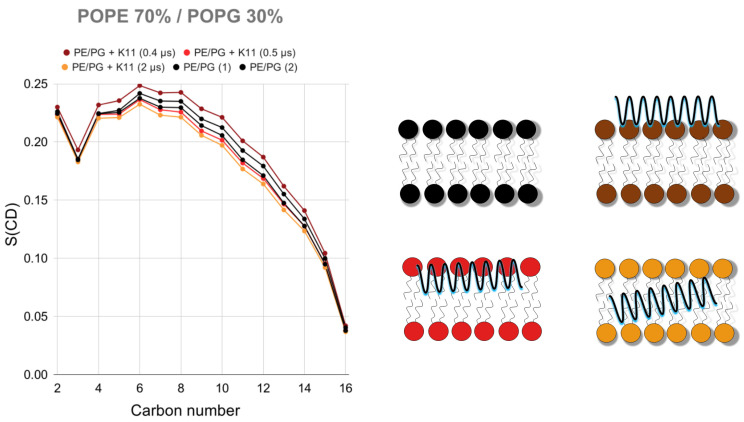
Order parameter of C-H moieties in palmitoyl side chains in membranes containing POPE (70%) and POPG (30%) as calculated from MD simulations in the absence (2 repetitions in black labeled as 1 and 2) and in the presence of K11 peptide. The order parameter varies in different ways along the 2 μs trajectory. The membrane is rigidified upon interaction (brown curve) but becomes more fluid as the peptide penetrates (red) and becomes internalized (orange curve).

The last step of the mechanism proposed in Figure 5 (step 4, helix flip and internalization) is essential because it explains the reduction of the lipid chain order experimentally demonstrated for the phospholipid acyl chains by the perturbation of ^2^H NMR spectra (Figure 3). It should be stressed that peptide anchoring (step 1 in Figure 5A) and membrane twisting (step 2 in Figure 5A) actually increase the order parameters of acyl side chains while the internalization (step 4 in Figure 5A) of the peptide in the hydrophobic core reduces it (Figure 6), as experimentally observed (Figure 3). The description of the proposed mechanism of action in four steps may require extending our 500 ns simulations further. The complete helix flip (step 4 in Figure 5A) is only observed in one of the three repetitions of the simulation with POPE/POPG membranes. We can hypothesize that in POPE/POPG mixtures (which better represent the bacterial membrane with respect to pure POPG) the activation energy for the penetration of the peptide is lower, thus allowing its detection in our 500 ns-long simulations. This is a reasonable hypothesis when considering that the PE headgroup has a smaller steric hindrance than that of PG and could facilitate the entrance of the peptide, as can also be rarely observed in simulations with pure POPE (Figure 4J). For this reason, we have extended the POPE/POPG calculation up to 2 μs.

It should be noted that the formation of the complex takes place in slow exchange in the NMR time scale (Figure 2A), meaning that k_ex_ << |Δω|. k_ex_ is the exchange rate constant (k_ex_ = k_on_[L] + k_off_ where k_on_ and k_off_ are the on and off-rate constants for the formation of the complex between the peptide P and the membrane M according to the equation P + M → PL) and |Δω| is the chemical shift difference between the free and the bound form of the peptide [73]. Our deviations are on the order of 0.2 ppm (Figure 2B), meaning that at 500 MHz |Δω| is ~600 s^−1^ (|Δω| = 2π|Δν|, where |Δν| is the chemical shift difference in Hz). Our slow exchange conditions therefore limit the value of k_ex_ and k_off_ to a maximum of 600 s^−1^ and the lifetime of the complex to a minimum of 1.7 milliseconds or much more, including the case of irreversible binding (the lifetime is the inverse of the off-rate constant). The detection of such long processes [74,75] would require more advanced sampling algorithms including dual-resolution MD [76], coarse-grain simulations, steered MD [77], umbrella-sampling [78,79], metadynamics [80,81], or replica exchange, among others [75,82,83]. This is beyond the scope of this work that aims at characterizing the steps at the very beginning of the interaction, in order to unravel the mode of action. The choice of all-atom MD allows us to directly compare the calculation with NMR data providing specific information on hydrogen and carbon atoms.

Peptide concentration plays an important role in the mechanism of action of AMPs because antimicrobials can act synergically to destabilize the target membrane using different strategies (carpet, pore formation by toroidal or barrel-stave models [76,84,85,86]). In order to confirm the hypothesis of an initial rigidification we calculated the order parameter for all membranes (Figure S8) increasing the number of peptides to simulate a higher concentration (one snapshot example of such calculation is shown in Figure 4I). Figure S8 shows a rigidification clearly visible for PG and PS membranes, as expected. The increase of order observed upon peptide binding is not so uncommon, and depends on factors like lipid composition of the membrane, temperature, and charge [58,60,87,88,89]. Sometimes, peptides that attach to the surface of the bilayer can increase acyl chain packing [90,91], especially when a strong electrostatic attraction is established [91]. The rigidification effect upon binding is also consistent with the observed hydrophobic thickness (Figure S9), that greatly increases for POPG, POPG containing membranes and CL as compared to POPC. Furthermore, the observed decrease in the electron density (Figure S9) can be a consequence of more water molecules being located near the polar head groups due to more loosely packaging caused by the presence of the peptides [88,92,93].

The effect of fluidification following the internalization was confirmed (Figure S10) by placing the peptide inside the bilayers at the beginning of the simulations (see example snapshots in Figure 4K,L). 

#### 2.3.3. K11 Approaches Phospholipids Head Groups from Opposite Leaflets Possibly Leading to Membrane Disassembly after Entering the Bilayer

The simulations of the fully internalized peptide can be thought of as a “prolongation” for those in which the peptide is able to access the membrane core. These simulations allow us to bypass the longer time scales needed to observe the full process. Two snapshots are shown in Figure 4K,L and they testify to a quite interesting phenomenon. The length of K11 helix is slightly shorter than the membrane thickness with the result that both the N-terminus and the C-terminus of K11 tend to recall polar head groups in the membrane core by binding with their oxygen atoms. Polar head groups on opposite leaflets almost come in close proximity. This is possible because polar head groups are initially grabbed by peripheral lysines residues and subsequently “walk” by detaching and attaching to the ones in the center of the helix. This is particularly evident in bacterial biomimetic membranes (Figure 4K,L). Once inside the bilayer, this mechanism would allow K11 to disassemble the membrane.

#### 2.3.4. PS Targeting Opens the Way to Possible New Biological Activities

The data presented in this work indicates that K11 destabilizes PS containing membranes (Figure 3 and Figure 4 but also Figures S5, S7 and S8), and this could be an indication of a possible anticancer activity, as already shown for some members [39] of its SR family (see Section 2.1 and Figure S1). It has to be noted that K11 was created as a combination of CP26 peptide (inspired by cecropin A1 and melittin) and a C-terminus from P18 peptide (inspired by cecropin A1 and magainin), which displays anticancer activity [20]. Similarly to what happens in apoptotic cells, cancerous cells tend to expose PS, a phospholipid normally found in the inner leaflet of the membrane [35]. A specific interaction with PS is probably the reason why a considerable number of antimicrobial peptides produced in eukaryotes (or inspired by them like K11) display anticancer activity while displaying low hemolytic activity and toxicity to healthy ones. Their eukaryotic origin explains their selectivity [94,95,96] As PS-targeting has proved to be effective as anti-cancer [36] or antiviral [97] therapies, the selective recognition of PS by K11 should not be undervalued.

One member of the K11 SR family also displays activity against fungi like *Candida albicans*. As shown in this work, K11 targets PS and PI, both being relevant for *Candida* virulence [44,45,46,47,48], together with PG [49].

## 3. Materials and Methods 

### 3.1. Synthesis of K11 Peptide

Fmoc(9-fluorophenylmethoxy)-amino acids, Fmoc-Tyr(tBu)-AC TentaGel^®^ resin (0.22 mmol/g, particle size: 90 µm) and Fmoc-TentaGel^®^-S RAM resin (0.24 mmol/g, particle size: 90 µm) were purchased from Iris Biotech (Germany). The other chemical compounds were purchased from VWR Chemicals, Iris Biotech or Acros and used without further purification. The peptides were synthesized on an CEM Liberty 1 Microwave Peptide Synthesizer, using standard automated continuous-flow microwave solid-phase peptide synthesis methods. Five-fold molar excess of the above amino acids was used in a typical coupling reaction. Fmoc-deprotection was accomplished by treatment with 20% (*v*/*v*) piperidine in *N*-methyl-2-pyrrolidone (NMP) at 75 °C. The coupling reaction was achieved by treatment with 2-(1*H*-benzotriazol-1-yl)-1,1,3,3-tetramethyluronium hexafluorophosphate (HBTU) and *N*,*N*-diisopropylethylamine (DIEA) in NMP using a standard microwave protocol (75 °C). The peptide was cleaved and side-chain deprotected by treatment of the peptide resin with a mixture of 1.85 mL of trifluoroacetic acid (TFA), 50 µL of triisopropylsilane, 50 µL H_2_O and 50 mg of DL-dithiothreitol, in respective percent proportions, 92.5/2.5/2.5/2.5, during 4 h at room temperature. The solid support was removed by filtration, the filtrate concentrated under reduced pressure, and the peptide precipitated from diethyl ether. The precipitate was washed several times with diethyl ether and dried under reduced pressure. The peptides were purified on an RP-HPLC C18 column (Phenomenex^®^ C18, Jupiter 4µ Proteo, 90 Å, 250 × 21.20 mm) using a mixture of aqueous 0.1% (*v*/*v*) TFA (A) and 0.1% (*v*/*v*) TFA in acetonitrile (B) as the mobile phase (flow rate of 3 mL/min) and employing UV detection at 210 and 254 nm. The purity of all peptides was found to be >95%.

K11 peptide was obtained as a white powder, with a total yield of 21.7%, after purification by reverse-phase HPLC (96% analytical purity) (see Figure S11). The concentration of the sample was determined by dissolving a precise amount of the powder in a precise volume of the buffer. The concentrated solution was subsequently divided in aliquots and lyophilized. Once redissolved in buffer, the concentration was confirmed by the absorbance at 280 nm, using a molar extinction coefficient of 5500 cm^−1^, M-1 (only one tryptophan is present) estimated by the ProtParam tool [98] of Expasy server (https://web.expasy.org/protparam/). 

### 3.2. Sequence Alignment by ADAPTABLE Web Server

The family of peptides sequence-related to K11 (KWKSFIKKLTKKFLHSAKKF) was created by the family generator page of ADAPTABLE webserver (http://gec.u-picardie.fr/adaptable/) using “Create the family of a specific peptide” option with the following parameters: “antibacterial = y”; “activity (µM) = 1”; “Substitution matrix = Blosum45”; “Minimum % of similarity = 51”. As ADAPTABLE continuously updates with new entries sequence-related families might change slightly with the time [38].

### 3.3. Sample Preparation, NMR Experiments and Analysis

Backbone and sequential resonance assignments were achieved by ^1^H,^13^C-HSQC, ^1^H,^1^H-TOCSY (mixing of 60 ms), and ^1^H,^1^H-NOESY (mixing of 200 ms) recorded on a 500 MHz Bruker spectrometer equipped with a 5 mm Broadband Inverse (BBI) probe. Deuterated sodium 3-(trimethylsilyl)propionate-d4 (TSP-d4) at a concentration of 100 µM was used as internal reference for chemical shift. Reference random coil values in our experimental conditions (*T* = 278 K, pH 6.6 and ionic strength 0.02 M) were calculated by POTENCY web server (https://st-protein02.chem.au.dk/potenci/) [99].

CD spectra were obtained in the far-UV (260–185 nm) on a J-815 Jasco spectropolarimeter (Tokyo, Japan). The CD measurement was performed at 5 °C, using a 1 mm path cell, with 5 accumulations for a 216.0 mg/mL sample in 10 mM sodium phosphate buffer, pH 6.6. All CD spectra measured were baseline corrected by subtracting the buffer spectrum.

A 1 mM sample of K11 (90% 10 mM phosphate buffer/10% D_2_O, pH 6.6) was titrated with a 1 M stock solution of DPC to a final DPC concentration of 60 mM. Titration was followed by 1D ^1^H-NMR at 278 K. For the assignment of the interacting form of the peptide 2D ^1^H,^1^H-NOESY and ^1^H,^13^C-HSQC were recorded at total DPC concentrations of 60 mM.

Bicelles were prepared as follows. A mixture of 33.3% DMPC and 66.7% DHPC in chloroform was used to obtain isotropic bicelles at a molar (q) ratio of 0.5. The solvent was evaporated under a nitrogen flow and the samples were then lyophilized and resuspended in a 10 mM phosphate buffer (pH 6.6) to reach a final concentration of 1 M (stock solution). DMPG, DMPS and DMPE containing bicelles were prepared as described above, except part of DMPC was replaced by DMPG (25%), DMPS (25%) or DMPE (10%) reproducing previous experiments [57]. A 1 mM sample of K11 (90% 10 mM phosphate buffer/10% D_2_O, pH 6.6) was titrated with bicelles up to a final lipid concentration of 70 mM and monitored at 278 K by a 1D ^1^H-NMR spectrum recorded after each addition.

MLVs containing deuterated palmitoyl chains were prepared according to the conventional protocol [100,101,102,103] using the following proportions: 50%:50% POPC/POPC:d31, 50%:50% POPG/POPG:d31, 50%:50% POPS/POPS:d31; 70%:30% POPE:d31/POPG, 50%:50% CL/POPC:d31, 70%:30% POPE:d31/POPC and 67%:27%:6% POPE:d31/POPG/CL. Lipids were solubilized in chloroform and solutions were mixed in order to obtain the right proportions in a total lipid amount of 60 mM. The resulting solution was evaporated under nitrogen gas flow. The sample was hydrated with ultrapure water, well-vortexed to promote a total hydration and lyophilized overnight to remove the traces of solvents. The resulting powder containing lipids was hydrated by 80 µL of ultra-pure water (for non-charged lipids) or 10 mM phosphate buffer pH 6.6 100 mM NaCl (for charged lipids), vortexed and homogenized using four free-thaw cycles involving one step of freezing (−80 °C, 15 min) followed by thawing (40 °C, 15 min) and shaking. Finally, the MLV samples were placed in a 7-mm ssNMR rotor to perform the experiments. 2.4 mM of peptide were added for interaction studies. 

ssNMR experiments were recorded at 310 K on a Bruker Avance Biospin 300 WB (7.05 T) equipped with a CP-MAS 7-mm probe (Bruker Biospin, Karlsruhe, Germany). Static ^2^H NMR was carried out applying a phase cycled quadrupolar echo pulse sequence (90°x-τ-90°y-τ-acq) [104]. The parameters used are listed below: spectral width of 150 kHz, π/2 pulse of 5.25 μs, an interpulse delay of 40 μs, a recycled delay of 1.5 s, and a number of acquisitions ranging from 8 k to 14 k depending on samples. For all spectra, an exponential line broadening of 100 Hz was applied before Fourier-transform from the top of the echo signal.

### 3.4. Molecular Dynamics Simulations

Systems for simulations were prepared using CHARMM-GUI [105,106,107]. A total of 128 lipid molecules were placed in each lipid bilayer (i.e., 64 lipids in each leaflet) and peptide molecules were placed over the upper leaflet at non-interacting distance (>10 Å). Lysine residues were protonated while histidine residue was protonated only on nitrogen in position δ. Initial peptide structure was obtained via I-TASSER [108] prediction tool, that produced a similar construct as the one produced by PEPFOLD [109,110] software. This structure was almost completely helical. Amidation of the C-terminus was achieved via the CHARMM terminal group patching functionality which is fully integrated in the CHARMM-GUI workflow. In case of calculations with eight peptides, they were placed next to each other but not in contact. A water layer of 50-Å thickness was added above and below the lipid bilayer which resulted in about 15,000 water molecules (30,000 in the case of CL) with small variations depending on the nature of the membrane. Systems were neutralized with Na^+^ or Cl^−^ counterions.

MD simulations were performed using GROMACS software [111] and CHARMM36 force field [112] under semi-isotropic (for bilayers) and isotropic (for micelles) NPT conditions [113,114]. The TIP3P model [54] was used to describe water molecules. Each system was energy-minimized with a steepest-descent algorithm for 5000 steps. Systems were equilibrated with the Berendsen barostat [115] and Parrinello-Rahman barostat [116,117] was used to maintain pressure (1 bar) semi-isotropically with a time constant of 5 ps and a compressibility of 4.5 × 10^−5^ bar^−1^. The Nose-Hoover thermostat [118,119] was chosen to maintain the systems at 310 K with a time constant of 1 ps. All bonds were constrained using the LINear Constraint Solver (LINCS) algorithm, which allowed an integration step of 2 fs. PBC (periodic boundary conditions) were employed for all simulations, and the particle mesh Ewald (PME) method [120] was used for long-range electrostatic interactions. After the standard CHARMM-GUI minimization and equilibration steps [113], the production run was performed for 500 ns (except when mentioned explicitly) and the whole process (minimization, equilibration and production run) was repeated once in the absence of peptide and twice in its presence. Convergence was assessed using RMSD and polar contacts analysis (see Figure S12).

All MD trajectories were analyzed using GROMACS tools [121,122] and Fatslim [123]. MOLMOL [124] and VMD [125] were used for visualization. Graphs and images were produced with GNUplot [126] and PyMol [127].

## 4. Conclusions

In this work, we have shown how the K11 peptide, largely unstructured in solution, assumes alpha helical conformation in the presence of biomimetic membranes. The interaction has very different consequences on the stability of the membrane depending on its nature. While PC and PE/PC bilayers are largely unaffected, PG, PS, PI and CL strongly interact with lysine residues. When examining bacterial-like mixtures containing both PG and PE, the large majority of the peptide-membrane interactions takes place with PG and the structurally related CL, if present. However, the same mechanism might well be active in the presence of PS, often exposed on the outer leaflet of cancer cells, which would suggest a potential anticancer activity of K11, as already described for its related peptides. The analysis of polar contacts reveals that lysine side chains tend to interact with oxygen atoms of the phosphate moiety (or the carboxylate of the serine in PS) rather than the OH of the glycerol or inositol head group, indicating that the recognition is based on the formation of salt bridges rather than H-bonds. This explains the lower affinity for PC and PE where the negative charge is neutralized by the choline and ethanolamine moieties, respectively. Once the salt bridges are formed, the peptide might penetrate as a screw, anchoring to the target with its N-terminus and twisting the membrane by further subsequent salt bridges involving pairs of lysine residues. The torque allows then the insertion of terminal hydrophobic side chains and eventually the internalization of the full peptide. Once inside, K11 can approach phospholipid head groups on opposite leaflets causing an effective disruption of the membrane potentially leading to the bacterial death. 

## Supplementary Materials

The following are available online at https://www.mdpi.com/1424-8247/14/1/1/s1, Figure S1: Sequence alignment of K11 peptide used as a bait in the ADAPTABLE web server; Figure S2: ^1^H,^13^C-HSQC spectral regions and assignment of K11 in solution and in the presence of DPC micelles, Figure S3: Minimum distance of each lysine side chain amine (atom name NZ) from membrane phosphorus atoms along the simulation trajectory of K11 interacting with DPC micelles, Figure S4: ^1^H NMR normalized spectra of K11 in the presence of DMPC/DHPC, DMPC/DHPC/DMPE, DMPC/DHPC/DMPG, and DMPC/DHPC/DMPS bicelles, Figures S5 and S6: Occurrence of polar atom contacts between K11 peptide and various membrane bilayers, Figure S7: Area per lipid in bilayers containing various phospholipids compositions as calculated from MD simulations in the presence of eight K11 peptides, Figure S8: Order parameter of C-H moieties in palmitoyl side chains in membranes containing various phospholipids compositions as calculated from multiple repetitions of MD simulations in the absence (2 repetitions in black labeled as 1 and 2) and in the presence (3 repetitions in red labeled from 1 to 3) of eight K11 peptides. The panel in the right bottom corner is an example of MD snapshot with POPE/POPG bilayer (color code in the caption of Figure 4). TOCL2 refers to CL, Figure S9: Electron density profiles for POPC, POPG and POPE/POPG/CL in presence of eight K11 peptides, Figure S10. Order parameter of C-H moieties in palmitoyl side chains in membranes containing various phospholipids compositions as calculated from multiple repetitions of MD simulations in the absence (2 repetitions in black labeled as 1 and 2) and in the presence (3 repetitions in red labeled from 1 to 3) of K11 peptide initially placed inside the bilayer. The panel in the right bottom corner is an example of MD snapshot with POPE/POPG/CL bilayer (color code in the caption of Figure 4). TOCL2 refers to C, Figure S11: Analytical purity of K11 peptide; Table S1: ^1^H and ^13^C NMR assignment of K11 peptide, Figure S12: Convergence analysis of the simulation of K11 peptide in the presence of POPE/POPG membrane. (A) Peptide RMSD (Cα carbon); (B) Polar contact block analysis in time intervals.
