# Supplementary material for: Antimicrobial Peptide K11 Selectively Recognizes Bacterial Biomimetic Membranes and Acts by Twisting Their Bilayers
Source: Pharmaceuticals (Basel). 2020 Dec 22;14(1):1. doi: 10.3390/ph14010001 (PMC7821925; doi:10.3390/ph14010001)
Supplement: Supplementary file 1 [file pharmaceuticals-14-00001-s001.pdf]

Supplementary Materials

Antimicrobial peptide K11 selectively recognises bacterial biomimetic membranes and acts by twisting their bilayers

Francisco Ramos-Martín, Claudia Herrera-León, Viviane Antonietti, Pascal Sonnet, Catherine Sarazin, and Nicola D'Amelio

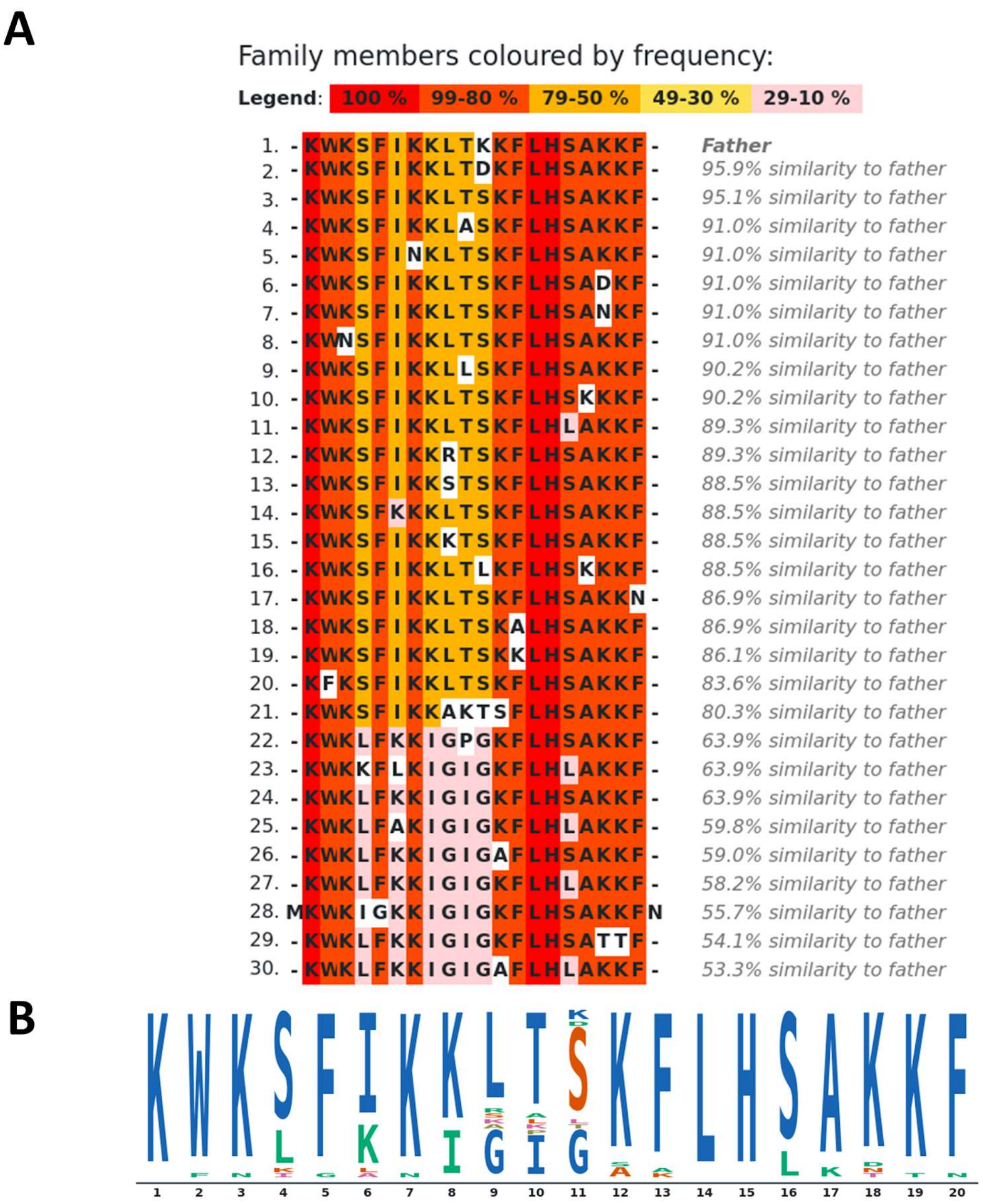

Activity of sequence-related antibacterial peptides from different sources (22-30)

22. Leukemia Cancer(IC50=100 µM), Lung Cancer(IC50=100 µM), Blood Cancer(IC50=75 µM), Breast Cancer(IC50=100 µM), Human, Escherichia coli KCTC 1682(MIC=3.125 µM), Salmonella typhimurium KCTC 1926(MIC=0.78 µM), Pseudomonas aeruginosa KCTC 1637(MIC=0.78 µM), Bacillus subtilis KCTC 1918(MIC=0.78 µM), Streptococcus pyogenes KCTC 3096(MIC=0.78 µM), Staphylococcus aureus KCTC 1621(MIC=3.125 µM), Staphylococcus aureus(MIC=3 µM), Escherichia coli(MIC=3 µM), S. typhimurium
23. Lung Cancer(IC50=2.6 µM), Lung Cancer(IC50=2.3 µM), Lung Cancer(IC50=1.8 µM)
24. Leukemia Cancer(IC50=65 µM), Lung Cancer(IC50=60 µM), Blood Cancer(IC50=30 µM), Breast Cancer(IC50=44 µM), Lung Cancer(IC50=15.8 µM), Lung Cancer(IC50=16.1 µM), Lung Cancer(MHC=10 µM), Lung Cancer(MHC=100 µM), Blood Cancer(MHC=10 µM), Blood Cancer(MHC=100 µM), Gastric Cancer(MHC=10 µM), Gastric Cancer(MHC=100 µM), Human, Human(MHC=25 µM), Human(MHC=100 µM), Escherichia coli KCTC 1682(MIC=3.125 µM), Salmonella typhimurium KCTC 1926(MIC=0.78 µM), Pseudomonas aeruginosa KCTC 1637(MIC=1.56 µM), Bacillus subtilis KCTC 1918(MIC=0.78 µM), Streptococcus pyogenes KCTC 3096(MIC=0.78 µM), Staphylococcus aureus KCTC 1621(MIC=3.125 µM), Escherichia coli ATCC 33694(MIC=6.25 µM), Bacillus subtilis ATCC 6633(MIC=1.56 µM), Human lung carcinoma NCI-H146(IC50=13 µM), Human lung carcinoma NCI-H128(IC50=9 µM), Human lung carcinoma NCI-H69(IC50=16 µM), Acinetobacter baumannii KCTC 2508(MIC=12.5 µM), Acinetobacter baumannii(MIC=12.5 µM), Streptococcus epidermidis KCTC 3096(MIC=3.12 µM), Candida albicans TIMM 1768(MIC=12.5 µM), Trichosporon beigelii KCTC 7707(MIC=6.25 µM), Escherichia coli CCARM 1229(MIC=8 µM), Escherichia coli CCARM 1229(MIC=32 µM), Pseudomonas aeruginosa 3552(MIC=8 µM), Pseudomonas aeruginosa 3552(MIC=4 µM), Staphylococcus aureus 3518(MIC=2 µM), Staphylococcus aureus 3518(MIC=64 µM), Staphylococcus aureus 1870(MIC=64 µM), Staphylococcus aureus 4716(MIC=4 µM), Staphylococcus aureus 3551(MIC=1 µM), Staphylococcus aureus(MIC=3 µM), Burkholderia pseudomallei, Escherichia coli(MIC=3 µM), Pseudomonas aeruginosa(MIC=2 µM), S. typhimurium
25. Lung Cancer(IC50=3.1 µM), Lung Cancer(IC50=2.4 µM)
26. Lung Cancer(IC50=9.3 µM), Lung Cancer(IC50=10.9 µM)
27. Lung Cancer(IC50=1.9 µM), Lung Cancer(IC50=1.3 µM), Lung Cancer(IC50=2.8 µM)
28. Bacillus subtilis ATCC 9372(MIC=100 µM), Bacillus anthracis Sterne 34F2(MIC=100 µM), Burkholderia thailandensis(MIC=50 µM)
29. Lung Cancer(IC50=36.2 µM), Lung Cancer(IC50=37.9 µM), Lung Cancer(IC50=47.7 µM)
30. Lung Cancer(IC50=2.9 µM), Lung Cancer(IC50=3.2 µM)

Figure S1. (A) Sequence alignment of K11 peptide used as a bait in the ADAPTABLE web server. (B) ADAPTABLE-generated consensus sequence and available data for the anticancer activity of each peptide of the family shown in (A).

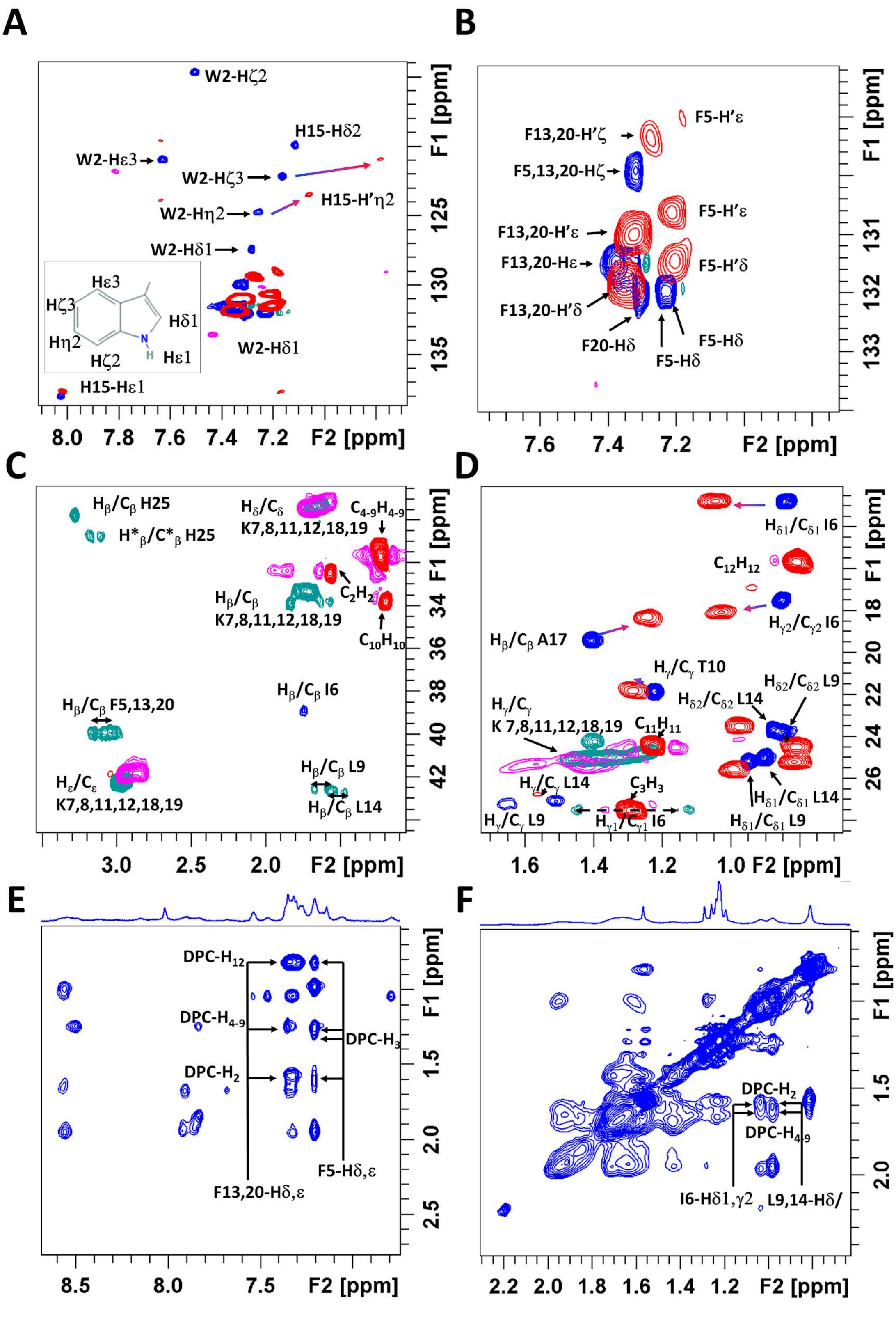

Figure S2. (A-D) $^{1}H,^{13}C$-HSQC spectral regions and assignment of K11 in solution (blue and green for positive and negative signals respectively) and in the presence of DPC micelles (red and magenta for positive and negative signals respectively): aromatic region (A), magnification of Phe aromatic region (B), Hβ region (C) and side chain regions (D). (E-F) NOESY spectrum of K11 in the presence of DPC micelles showing meaningful NOEs in the aromatic/aliphatic (E) and amide (F) regions.

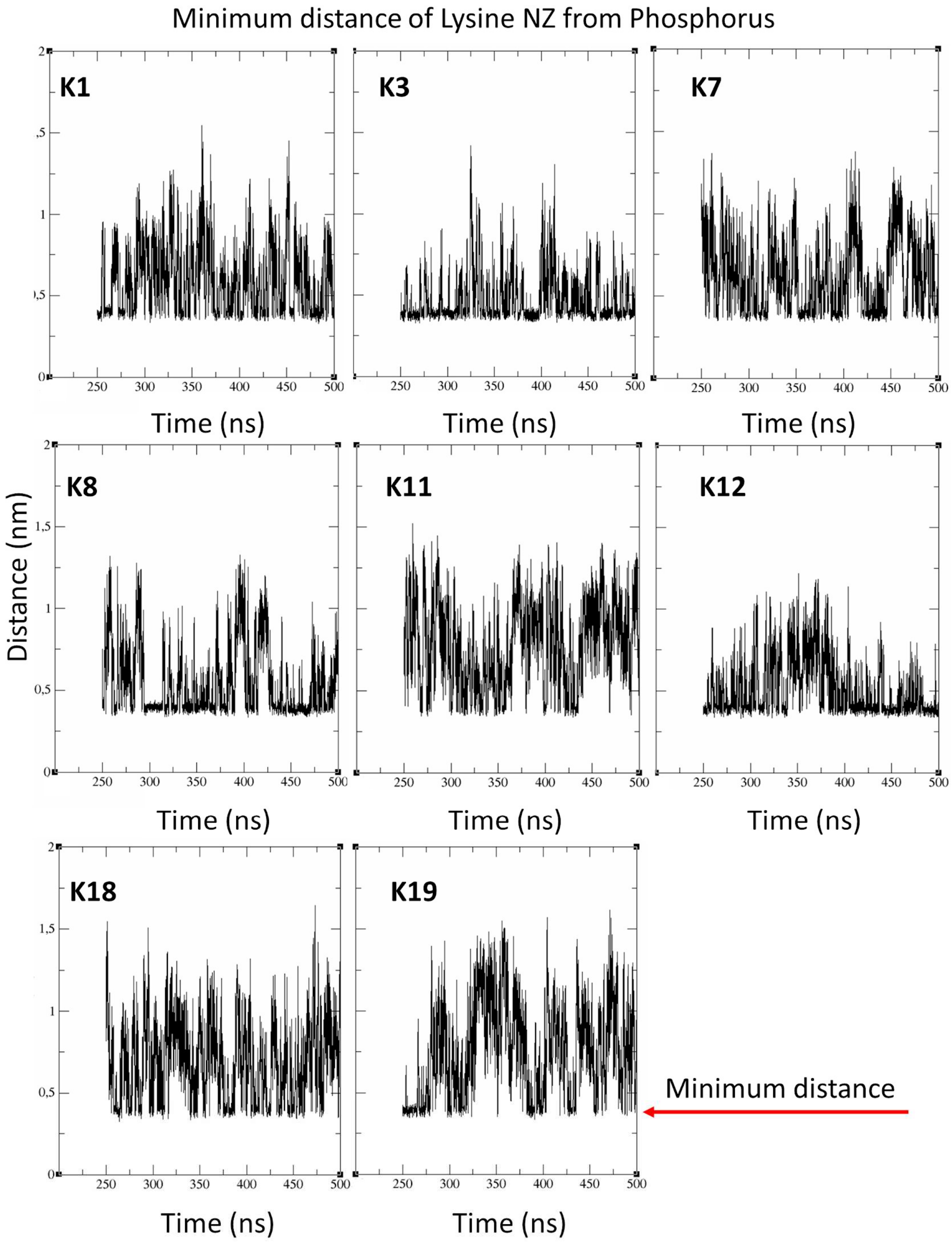

Figure S3. Minimum distance of each lysine side chain amine (atom name NZ) from membrane phosphorus atoms along the simulation trajectory of K11 interacting with DPC micelles.

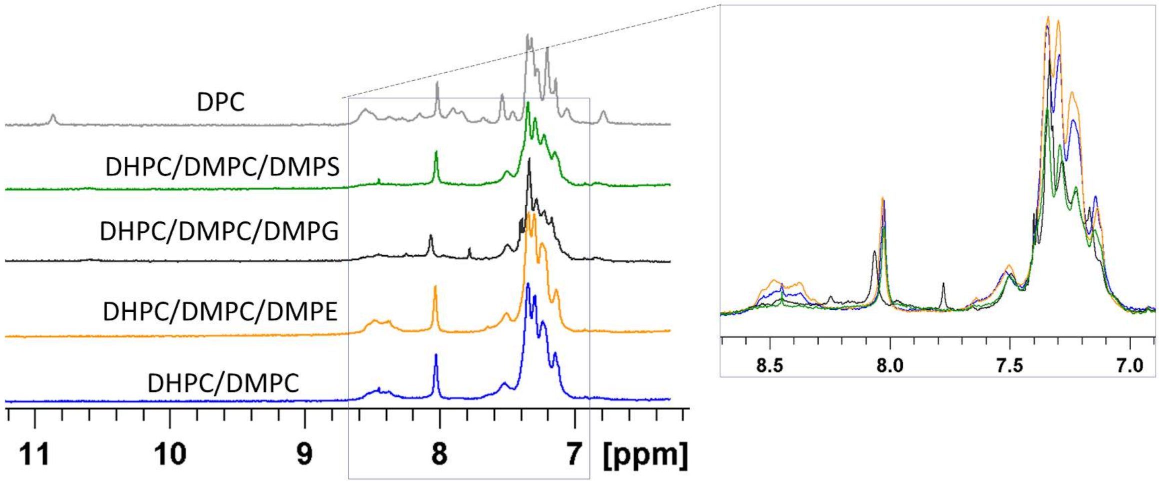

Figure S4. ^{1}H NMR normalized spectra of K11 1mM in the presence of DMPC/DHPC (blue), DMPC/DHPC/DMPE (orange), DMPC/DHPC/DMPG (black), DMPC/DHPC/DMPS (green) bicelles at a total concentration 70 mM. The spectrum in the presence of DPC 60 mM (gray) is shown for comparison.

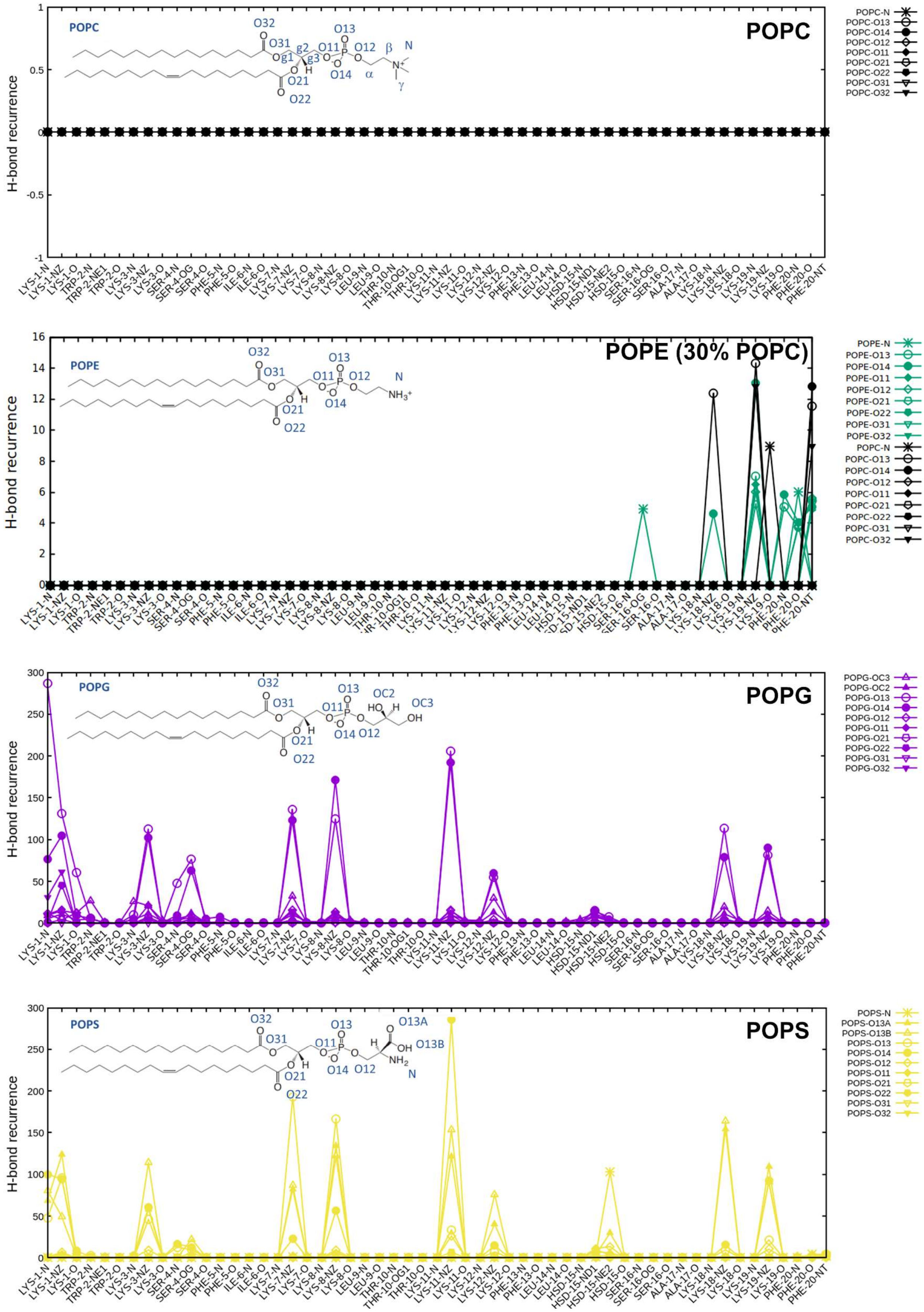

Figure S5. Occurence of polar atom contacts (H-bonds and salt bridges) between K11 peptide and various membrane bilayers calculated along MD simulation trajectories.

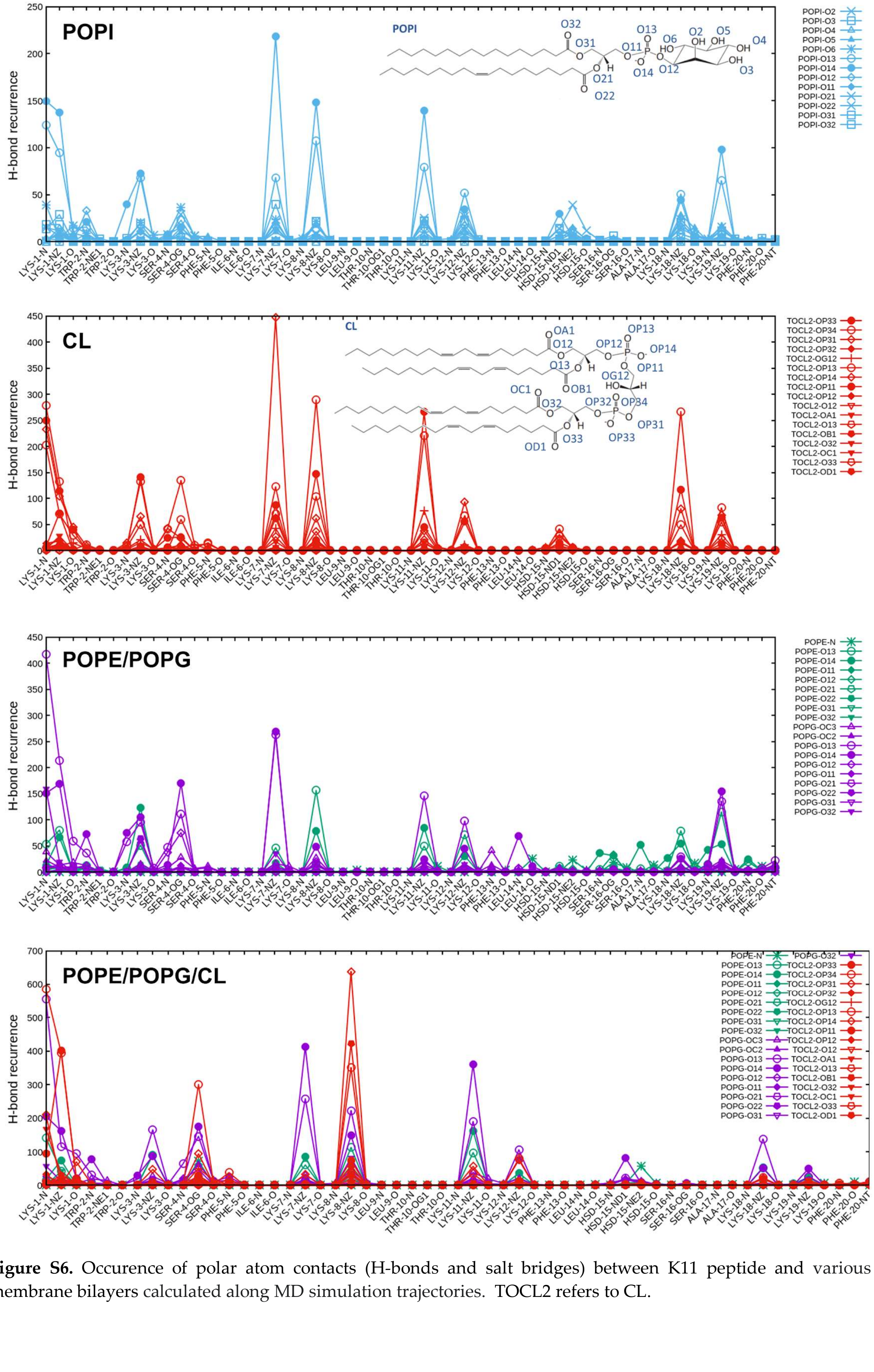

Figure S6. Occurence of polar atom contacts (H-bonds and salt bridges) between K11 peptide and various membrane bilayers calculated along MD simulation trajectories. TOCL2 refers to CL.

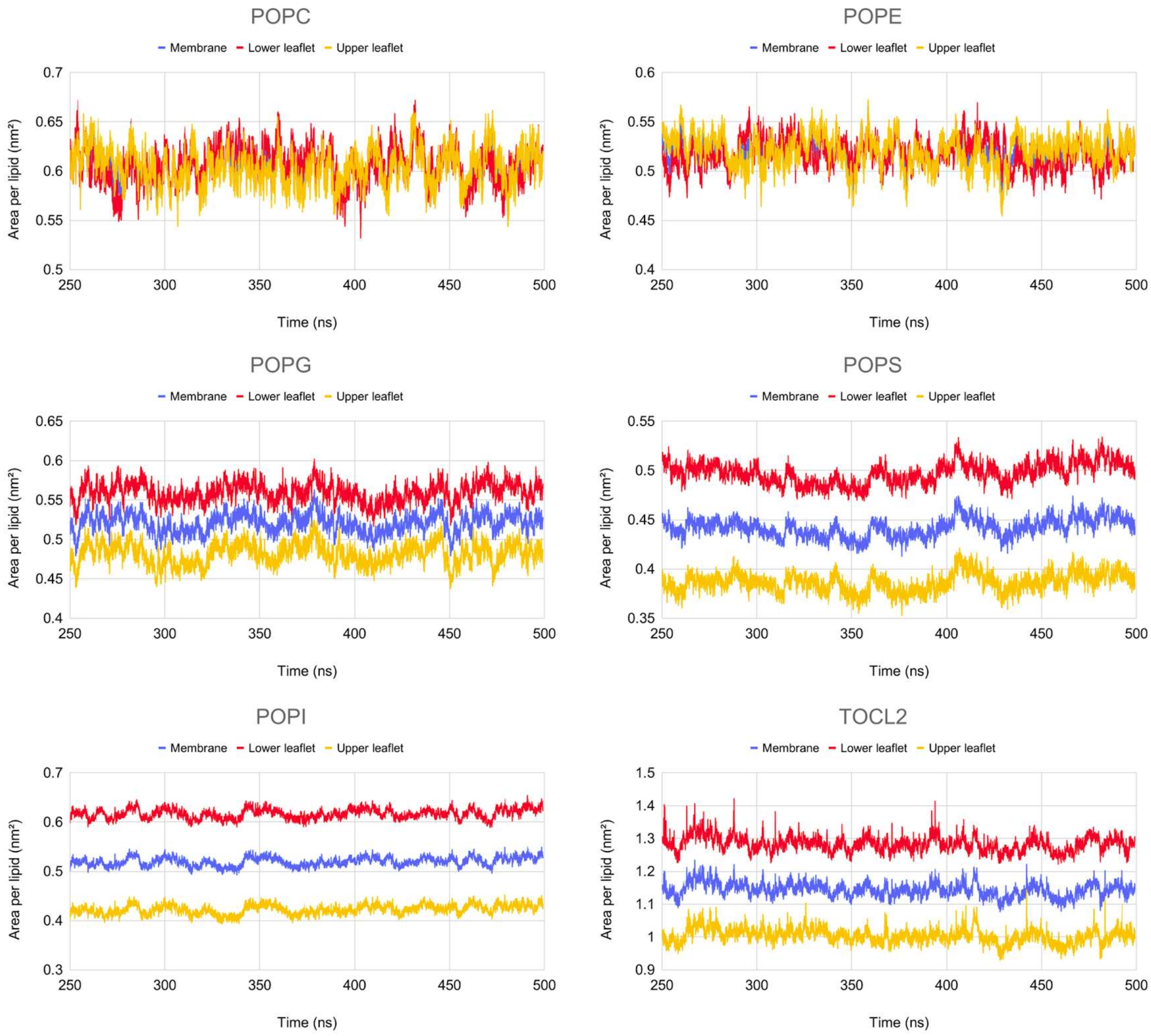

Figure S7. Area per lipid (nm^2) in bilayers containing various phospholipids compositions as calculated from MD simulations in the presence of eight K11 peptides. The average value is shown in blue while the upper and lower leaflet are shown in yellow and red respectively. TOCL2 refers to CL.

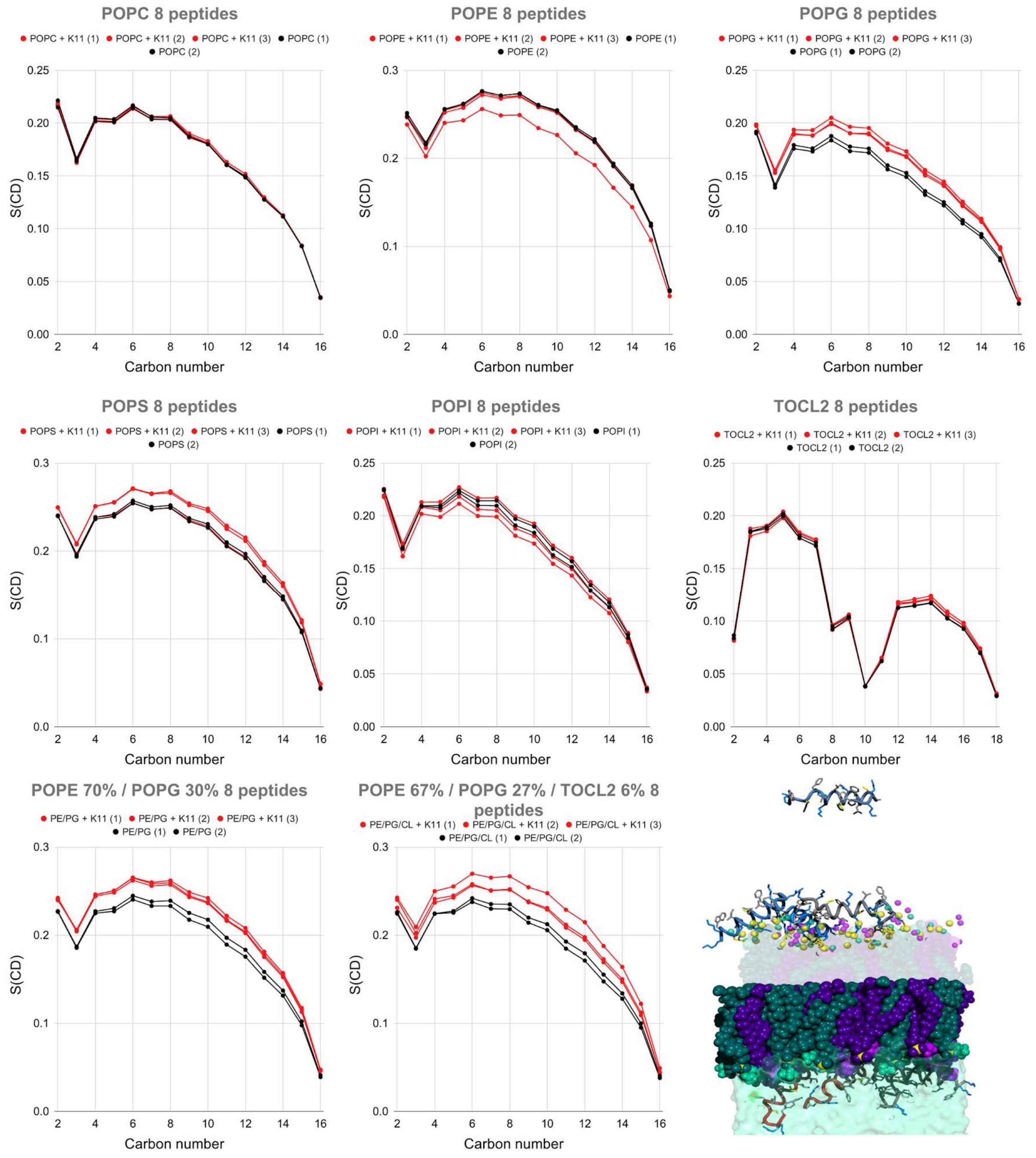

Figure S8. Order parameter of C-H moieties in palmitoyl side chains in membranes containing various phospholipids compositions as calculated from multiple repetitions of MD simulations in the absence (2 repetitions in black labeled as 1 and 2) and in the presence (3 repetitions in red labeled from 1 to 3) of eight K11 peptides. The panel in the right bottom corner is an example of MD snapshot with POPE/POPG bilayer (color code in the caption of Figure 4). TOCL2 refers to CL.

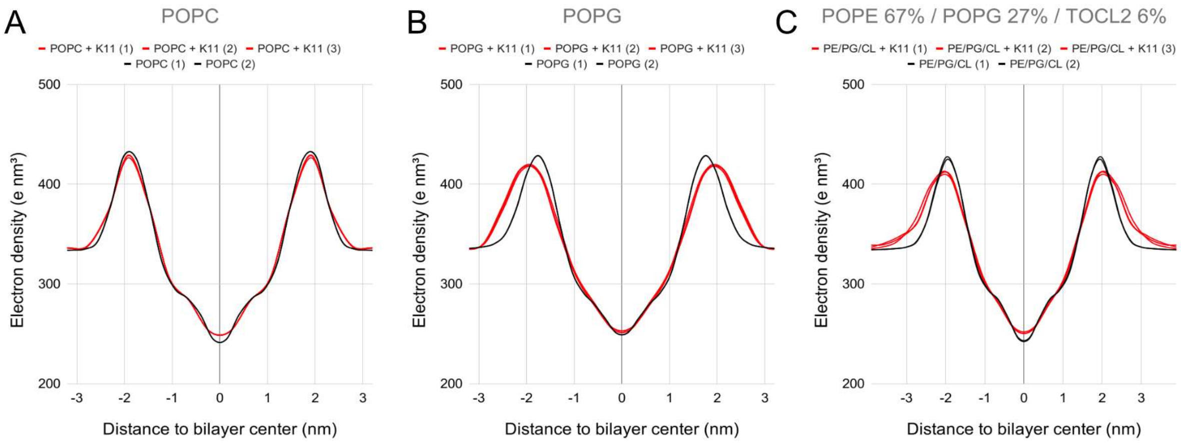

Figure S9. Electron density profiles for POPC (A), POPG (B) and POPE/POPG/CL (C) in presence of eight K11 peptides. TOCL2 refers to CL.

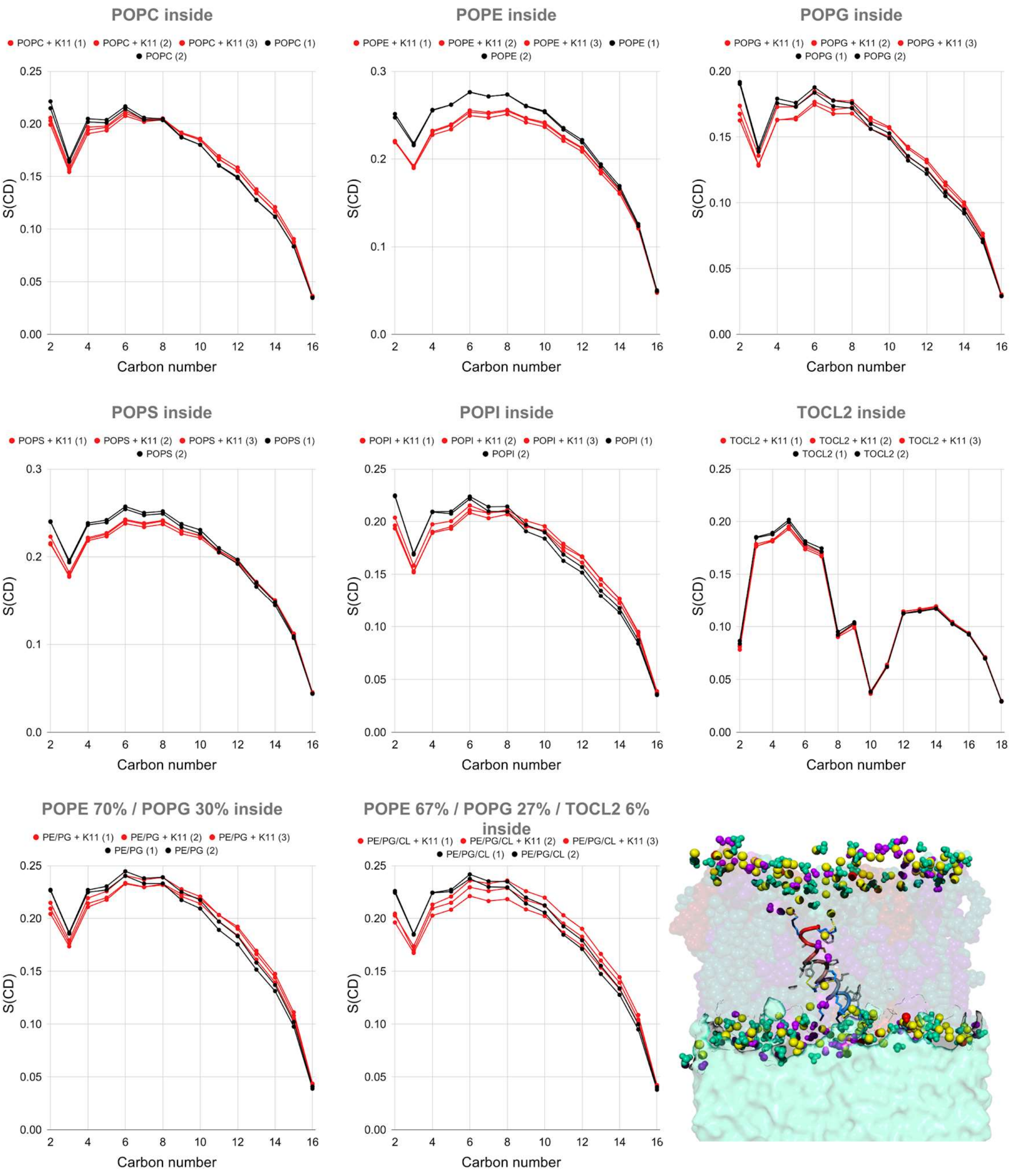

Figure S10. Order parameter of C-H moieties in palmitoyl side chains in membranes containing various phospholipids compositions as calculated from multiple repetitions of MD simulations in the absence (2 repetitions in black labeled as 1 and 2) and in the presence (3 repetitions in red labeled from 1 to 3) of K11 peptide initially placed inside the bilayer. The panel in the right bottom corner is an example of MD snapshot with POPE/POPG/CL bilayer (color code in the caption of Figure 4). TOCL2 refers to CL.

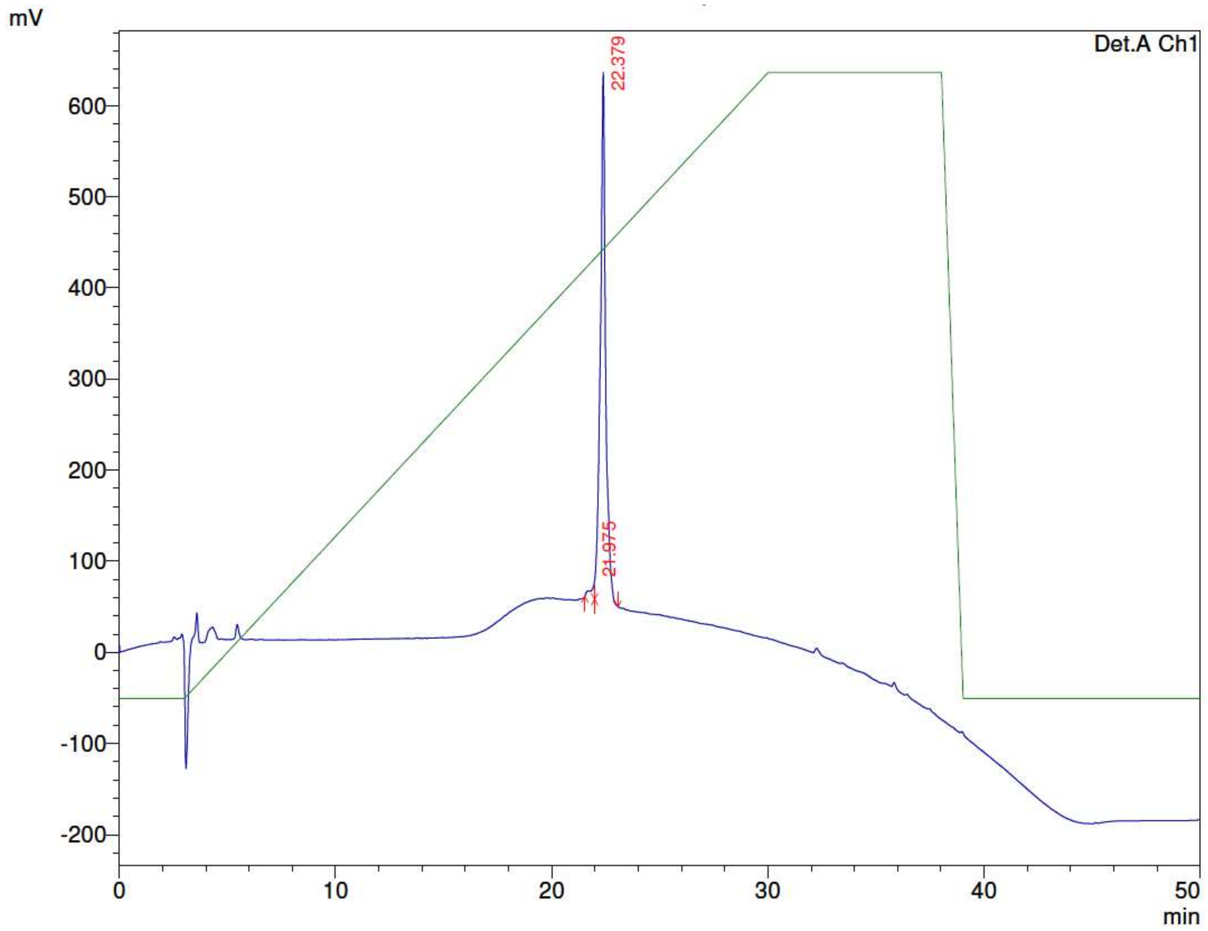

Detector A Ch1 210nm

Peak Purity Index	Ret. Time	Area	Height	Area %	Height %
	21.975	222959	18837	2.187	3.139
	22.379	9970525	581156	97.813	96.861
		10193484	599992	100.000	100.000

Figure S11. Analytical purity of K11 peptide. HPLC C12 column (Phenomenex® C12, Jupiter 4µ Proteo, 90 Å, 250x4.6 mm) using a mixture of aqueous 0.1% (v/v) TFA (A) and 0.1% (v/v) TFA in acetonitrile (B) as the mobile phase and employing UV detection at 210 nm.

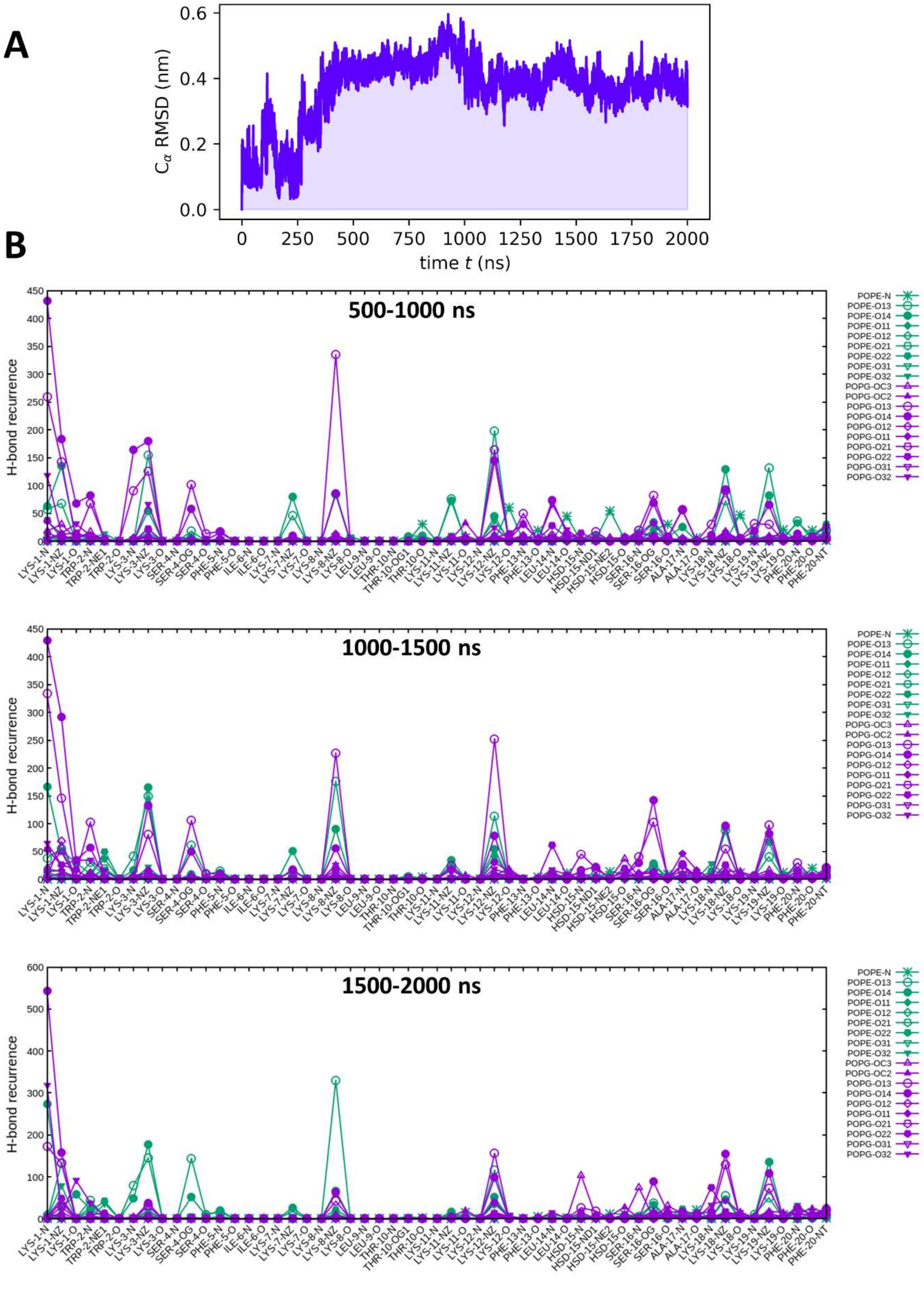

Figure S12. Convergence analysis of the simulation of K11 peptide in the presence of POPE/POPG membrane. (A) Peptide RMSD (Cα carbon); (B) Polar contact block analysis in time intervals.

Table S1. ^{1}H and ^{13}C NMR assignment of K11 peptide 1 mM in 10 mM phosphate buffer (10% D_2O), pH 6.6, 278K

	LYS1	TRP2	LYS3	SER4	PHE5	ILE6	LYS7	LYS8	LEU9	THR10
^{1}H	NH=X	NH=8.165	NH=8.17	NH=8.13	NH=8.395	NH=8.146	NH=8.48	NH=8.384	NH=8.56	NH=8.3
	α =3.914	α =4.63	α =4.16	α =4.188	α =4.63	α =4.06	α =4.24	α =4.2	α =4.45	α =4.325
	β_1=1.818	β =3.28	β_1=1.6	β =3.75	β_1=3.04	β =1.75	β =1.76	β =1.7	β =1.57	β =4.2
	β_2 =1.62	ϵ_1=10.23	β_2 =1.56		β_2=3.09	γ_1=1.75	γ =1.45	γ =1.71	γ =1.65	β_2=2.90
	γ =1.395	ϵ_3 =7.62	γ=1.24		δ =7.23	γ_2=0.86	δ_1=1.45	δ =1.36	δ_1=0.87	δ_2=1.2
	δ= 1.7	δ =7.28	δ=1.62		ϵ =7.4	δ =0.85	δ_2=1.13	ϵ =?	δ_2=0.95	
	ϵ =2.97	ζ_2=7.5	ϵ =?		ζ =7.4		ϵ =3.07			
		ζ_3=7.17								
		η_2 =7.25								
^{13}C	α =55.94	α =58	α=56.14	α =58.25	α =57.9	α =61	α =56.5	α =56.5	α =55.17	α =62.1
	β =33.93	β =29.9	β=33.87	β =64	β =40	β =38.5	β =33.2	β =33.2	β =42.58	β =70.2
	γ =24.91	ϵ_3 =120	γ =24.7		δ =132	γ_1 =33.4	γ =25	γ =25	γ =27.17	γ_2=21.81
	δ =29.44	δ_1 =127	δ=29.13		ϵ =131	γ =17.45	δ =27.5	δ =29.5	δ_1 =23.65	
	ϵ =42.2	ζ_2 =114.4	ϵ =?		ζ =130	δ_1 =12.8	ϵ =42.2	ϵ =42.2	δ_2=25.15	
		ζ_3 =122								
		η_2 =124								

	LYS11	LYS12	PHE13	LEU14	HIS15	SER16	ALA17	LYS18	LYS19	PHE20
^{1}H	NH=8.54	NH=8.44	NH=8.46	NH=8.3	NH=8.43	NH=8.45	NH=8.54	NH=8.44	NH=8.54	NH=8.42
	α =4.24	α =4.25	α =4.59	α =4.32	α =4.6	α =4.4	α =4.33	α =4.25	α =4.24	α =4.58
	β =1.74	β =1.7	β_1 =3.08	β =1.54	β =3.17	β =3.85	β =1.4	β =1.7	β =1.74	β_1 =3.04
	γ =1.4	γ =1.315	β_2=2.98	γ =1.5	γ_2 =7.11			γ =1.315	γ =1.4	β_2 =3.14
	δ =1.67	δ =1.68	δ =7.21	δ_1 =0.85	ϵ_1 =8.07			δ =1.68	δ =1.67	δ =7.3
	ϵ =?	ϵ =3.1	ϵ =7.4	δ 2 =0.88				ϵ =3.1	ϵ =?	ϵ =7.4
			ζ =7.4							ζ =7.4
^{13}C	α =56.56	α =56.5	α =57.7	α =55.1	α =56.5	α =58.4	α =52.7	α =56.5	α =56.56	α =57.77
	β =33.2	β =33.3	β =40	β =42.7	β =30.74	β =64	β =19.41	β =33.3	β =33.2	β =40
	γ =25	γ =25	δ =132	γ =27.05	γ 2 =119			γ =25	γ =24.24	δ =132
	δ =29.3	δ =29.35	ϵ =131	δ_1 =23.64	ϵ 1 =137			δ =29.3	δ =29.3	ϵ =131
	ϵ =42.2	ϵ =42.2	ζ =130	δ_2 =23.77				ϵ =42.2	ϵ =42.2	ζ =130
